# Bioactive Molecular Networking for Mapping the Antimicrobial Constituents of the Baltic Brown Alga *Fucus vesiculosus*

**DOI:** 10.3390/md18060311

**Published:** 2020-06-13

**Authors:** Larissa Buedenbender, Francesca Anna Astone, Deniz Tasdemir

**Affiliations:** 1 GEOMAR Centre for Marine Biotechnology (GEOMAR-Biotech), Research Unit Marine Natural Products Chemistry, GEOMAR Helmholtz Centre for Ocean Research Kiel, Am Kiel-Kanal 44, 24106 Kiel, Germany; lbuedenbender@geomar.de (L.B.); francesastone@gmail.com (F.A.A.); 2 Faculty of Mathematics and Natural Sciences, Kiel University, Christian-Albrechts-Platz 4, 424118 Kiel, Germany

**Keywords:** *Fucus vesiculosus*, brown alga, bioactive molecular networking, in silico dereplication, metabolomics, antimicrobial, *Staphylococcus aureus*, galactolipid, MGDG, phlorotannin

## Abstract

The brown alga *Fucus vesiculosus* is common to the intertidal zones of the Baltic Sea, where it is exposed to high fouling pressures by microorganisms. Our previous studies showed, repeatedly, the consistent antimicrobial activity of *F. vesiculosus* crude extracts against human pathogens, while untargeted metabolomics analyses have revealed a variety of metabolites. In this study, we applied the UPLC-QToF-MS/MS-based “bioactive molecular networking” (BMN) concept on the most bioactive *n-*hexane and *n*-butanol subextracts of Baltic *F. vesiculosus* coupled with in silico dereplication tools to identify the compounds responsible for antimicrobial activity. The first antimicrobial cluster identified by BMN was galactolipids. Our targeted isolation efforts for this class led to the isolation of six monogalactosyldiacylglycerol (MGDG) derivatives (**1**–**6**) and one digalactosyldiacylglycerol (DGDG, **7**). The MGDGs **5** and **6** and the DGDG **7** exhibited activity against *Staphylococcus aureus.* The second compound class with high bioactivity was phlorotannins. In particular, phlorethol-type phlorotannins showed high correlations with antimicrobial activity based on the BMN approach, and two phlorotannins (**8**–**9**) were isolated. This study shows that antimicrobial components of *F. vesiculosus* reside in the algal cell walls and membranes and that BMN provides a complementary tool for the targeted isolation of bioactive metabolites.

## 1. Introduction

The edible brown seaweed *Fucus vesiculosus* (phylum: Ochrophyta), also known as bladder wrack, is widely distributed and abundant in the intertidal zones of the Northern hemisphere; in particular, in the North and Baltic Sea, Atlantic coasts of Europe and North America. Small populations of bladder wrack are also found on the East Pacific coast of Canada [1]. As seawater contains approximately one million bacteria per millilitre, marine seaweeds are exposed to immense fouling pressure [2,3]. Diverse microorganisms attach to algal surfaces to form biofilms that can have beneficial but also adverse effects to the seaweed. Uncontrolled fouling affects algal growth, reproduction and susceptibility to diseases [4]. In order to thrive in the marine environment, macroalgae have evolved both mechanical and chemical defence strategies to control microfouling [4]. Our previous investigations on the surface microbiome and metabolome of *F. vesiculosus* pointed out the potential contribution of microbial epibionts in antifouling chemical defence of their hosts by producing antimicrobial compounds [5]. However, in addition, truly algal metabolites have been proposed to be involved in antimicrobial defence of seaweeds [6]. Due to ecological fouling pressures, macroalgae produce a broad range of structurally diverse and biologically active metabolites, which also provide a potential source for drug leads to fight pathogens causing infections in human [7,8].

The antimicrobial potential of brown seaweeds has been widely reported in the literature. This includes, in particular, high inhibitory effects against Gram-positive bacteria such as *Staphylococcus aureus* and *Bacillus subtilis* [6]. Brown algal metabolites with reported antimicrobial activities include phenolic compounds, carotenoids, polysaccharides and lipids [6]. The reported antimicrobial activity of *F. vesiculosus* is generally on crude extracts and fractions, while only very few studies have tested pure compounds derived from this seaweed. A polyhydroxylated fucophlorethol isolated from Norwegian *F. vesiculosus* showed inhibition of *Escherichia coli* and *S. aureus* [9], while the carotenoid fucoxanthin inhibited settlement of four marine bacteria at its natural concentrations of 0.3 to 10 mg/cm^2^ on the algal surface [10]. Our investigations on the seasonal variations in the metabolome and bioactivity profile of Baltic *F. vesiculosus* showed consistent activity of organic crude extracts against Methicillin-resistant *Staphylococcus aureus* (MRSA) throughout the year, while abundance of certain classes of compounds varied depending on the sampling month [8]. For instance, the abundance of phlorotannins was highest in summer but lowest in winter when production of lipid and chlorophyll-type compounds was upregulated. This indicates that antimicrobial activity cannot be attributed to a single compound or class, but potentially a range of different metabolites. Thus, the specific origin of the antimicrobial activity in *F. vesiculosus* remains to be elucidated.

Untargeted tandem mass spectrometry spectral mining has become one of the most favoured approaches in metabolomics studies. The Global Natural Products Social Molecular Network (GNPS) platform provides an open-access tool that uses an algorithm to compare large numbers of MS/MS spectra based on their cosine similarities [11]. Related ions with similar MS/MS fragment patterns that cluster together can be visualized in molecular networks (MN). Furthermore, the tool enables automated dereplication of the experimental fragment spectra against the GNPS database facilitating easy annotation of compound classes in the network. In classical MN, raw MS/MS spectra files are uploaded directly to the GNPS webserver to generate a MN; this method often includes multiple nodes for one compound if detected over a large retention time span, as well as their isomers and generates massive MNs that are not fully representative of the actual number of compounds in a sample. Therefore, a second method termed Feature-based Molecular Networking (FBMN) has been offered in the GNPS environment [12], where a feature detection and alignment tool (MZmine, OpenMS, XCMS, etc.) is used to pre-process the LC-MS/MS data. Data can be filtered for noise, duplicate peaks and isomers, thus allowing relative quantification within the FBMN. It is furthermore possible to integrate biological activity data into FBMN and a complementary workflow termed “bioactive molecular networking” (BMN) has recently been described [13]. Thereby, features of chromatographic profiles of a fractionated extract derived through, e.g., MZmine are correlated with the bioactivity levels of the respective fractions. The bioactivity score is based on the statistical Pearson correlation coefficients between chemical features and observed bioactivity and can be mapped out on the molecular network to predict potential bioactive compounds or chemical families and thus guiding their targeted isolation. Herein, we applied the BMN concept in order to map the antimicrobial components of the *F. vesiculosus* metabolome and prioritize them for rapid and targeted isolation and chemical characterization.

## 2. Results

The algal material was extracted with MeOH and subjected to a modified Kupchan partition scheme [14] to yield *n*-hexane, DCM, *n*-BuOH and aqueous subextracts. Inhibitory activity of all subextracts against MRSA was evaluated at a test concentration of 100 µg/mL. The highest antimicrobial activity was tracked in the *n*-hexane (80% inhibition) and *n*-BuOH (75% inhibition) subextracts, whereas minimal inhibition was observed in the DCM (< 20%) and aqueous subextracts (39%) (Figure S1). In order to identify the compounds responsible for their antimicrobial activity, we employed bioactive molecular networking (BMN) on bioactive subextracts [13]. The *n*-hexane subextract was further fractionated by SiO_2_ flash chromatography to yield 40 fractions (H8–H48), while the *n*-BuOH subextract was fractionated into 29 fractions (B1–B29) by preparative C18 HPLC. All subfractions were tested against MRSA (at 100 µg/mL) and profiled by tandem UPLC-QToF-MS/MS. After MZmine pre-processing of the UPLC-QToF-MS/MS data, the FBMN workflow was implemented through the GNPS platform [12]. The Pearson correlation coefficients (*r*) were calculated between the relative abundances of the molecular ions (peak area) in the fractions and their MRSA-bioactivity levels in order to obtain a bioactivity score for each compound. Ions with positive bioactivity scores closest to 1 present the most promising target compounds. The generated BMNs were imported to Cytoscape where nodes were coloured based on their bioactivity levels and molecular ions with high bioactivity scores were visualised as larger nodes.

### 2.1. Bioactive Molecular Netwoking of the n-Hexane Subextract

**Figure 1:**
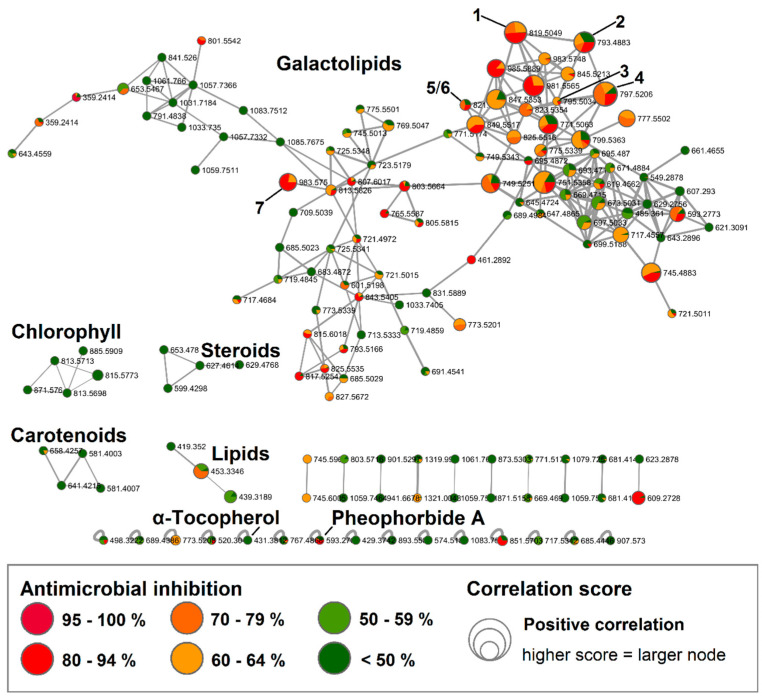
Bioactive molecular networking of the fractionated *F. vesiculosus n*-hexane subextract displaying compounds, in particular galactolipids, with high bioactivity scores. Nodes are coloured according to their MRSA inhibitory activity (%) at 100 µg/mL. The node size reflects the bioactivity scores/Pearson correlations of the ions. Nodes of isolated compounds **1**–**7** are annotated in the network. (Data recorded in positive ionization mode).

Out of 40 fractions obtained from the *F. vesiculosus n*-hexane subextract, eight fractions (H8, H16, H41-42, H45-48) showed anti-MRSA inhibition above 70% at 100 µg/mL (Figure S1). For BMN, UPLC-QToF-MS/MS data were acquired for all 40 fractions in positive mode. The obtained BMN consisted of 145 nodes and displayed five main molecular clusters (Figure 1). Through the standard FBMN workflow, only one single node—the vitamin α-tocopherol (*m/z* 431.3812 [M + H]^+^)—could be dereplicated against the GNPS library (Figure S2). Consequently, we enhanced our dereplication success via the implementation of in silico MS/MS database matching [15] and manual dereplication to annotate 29 nodes to known compounds (Table S1). The in silico database matching allowed for the annotation of one node of the main network cluster to (2*S*)-1,2-*bis*-*O*-eicosapentaenoyl-3-*O*-*β*-d-galactopyranosyl-*sn*-glycerol (*m/z* 823.5354 [M + H]^+^), revealing the predominant constituents of this subextract to be galactolipids, a compound class commonly found in marine algae, including *Fucus* species. Comprehensive manual dereplication of the UPLC-QToF-MS/MS data of the fractions allowed for the annotation of another 23 nodes in this compound class, in particular 19 monogalactosyldiaclyglycerols (MGDGs), two digalactosyldiaclyglycerols (DGDGs) and three digalactosylmonoaclyglycerols (DGMGs) that all showed different polyunsaturated substitutions of eicosanoic acid, octadecanoic acid, hexadecanoic acid or tetradecanoic acid (Table S1 and Figure S3). The highest bioactivity scores (*r* = 0.3–0.37, *p* < 0.05) were associated with several nodes of the galactolipid cluster (indicted by larger node size in Figure 1). The degree of the positive Pearson correlation coefficients for these nodes were statistically ranked as moderate [16].

Two chlorophyll-type compounds, pheophytin A and B (*m/z* 871.5760 [M + H]^+^ and *m/z* 885.5909 [M + H]^+^, respectively), were matched to a small cluster without detected bioactivity (Figure 1). Using the in silico MS/MS database, another single node was annotated as the related chlorophyll-type compound, pheophorbide A (*m/z* 593.2769 [M + H]^+^). Two more clusters were putatively annotated to a steroid and a lipid cluster, natural product classes that are expected to be present in a nonpolar *n-*hexane extract. Nodes of these clusters gave various matches to these compound classes in the in silico database, but no specific compound was identified based on the MS/MS fragmentation data. Furthermore, the characteristic brown algal carotenoid fucoxanthin in its dehydrated form (*m/z* 641.4215 [M − H_2_O + H]^+^) was detected in a separate carotenoid cluster (Figure 1 and Table S1). None of the latter four clusters showed positive correlations with anti-MRSA activity.

### 2.2. Bioactive Molecular Networking Guided Isolation of Galactolipids

**Figure 2:**
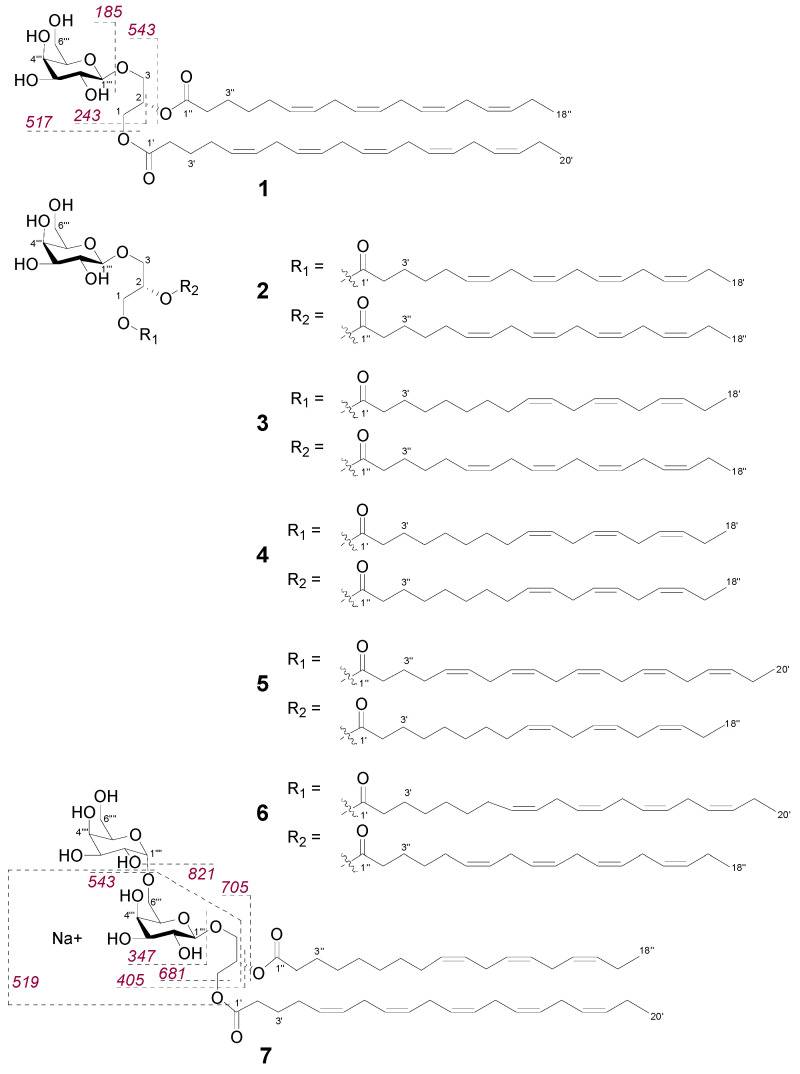
Structures of galactolipids (**1**–**7**) isolated from Baltic *F. vesiculosus*. Indicative MS/MS fragmentation ions are labelled for the [M + Na]^+^ ions of **1** and **7**. (Spectral data inclusive labelled fragmentation pathways of **2**–**6** can be found in supplementary Figures S8–S17).

The initial BMN and dereplication analysis revealed a set of galactolipids that positively correlated with antimicrobial activity in the *n*-hexane subextract (Figure 1). Thus, we targeted the isolation and structure elucidation of a range of galactolipids (Figure 2). We also aimed to study structure activity relationships (SARs) for this class and whether the SARs were reflected in nodes of the BMN. MGDGs, as well as DGDGs, have a glycerol backbone to which one or two polyunsaturated fatty acid chains of different lengths and degree of unsaturation are linked at the *sn*-1 and *sn*-2 positions and a polar headgroup of one or two galactose units is connected to *sn*-3 [17]. The prefix “*sn*” (for stereospecifically numbered) is used to designate the configuration of the glycerol derivatives, where the carbon that would “appear on top in the Fischer projection shows a vertical carbon chain with the hydroxyl group at C-2 to the left” is assigned as C-1 [18].

The galactolipids were predominantly found in the fractions H39-H42 that showed inhibition of MRSA between 65–85% (Figure S1). Based on HPLC–Evaporative Light Scattering Detector (ELSD) analysis, compound **1** appeared as the major MGDG in the *n*-hexane bioactive subfractions and was isolated from the combined fractions H39-H42. HR-MS data of **1** revealed a sodium adduct ion at *m/z* 819.5018 [M + Na]^+^ that corresponded to the molecular formula C_47_H_72_O_10_Na. HR-MS/MS fragmentation analysis showed two high intensity fragment ions at *m/z* 517.2783 [M + Na]^+^ and *m/z* 543.2935 [M + Na]^+^ (Figure S4) that were indicative of the loss of C20:5 (carbon atoms:double bonds) and a C18:4 acyl chain, respectively.

According to Guella et al. [19], the regiochemical positions of the two acyl chains can be established based on the fragment ion intensity, as the loss of the acyl chain at position *sn*-1 produces a more intense peak than the one from the loss of the acyl chain at position *sn*-2. Hence, the C20:5 acyl chain in **1** was determined to be at position *sn*-1 and C18:4 at *sn*-2. We confirmed the structure of the MGDG **1** and its relative stereochemistry with a set of 1D and 2D NMR experiments (Table 1), which also assisted the structure elucidation of the remaining galactolipids that are assumed to originate from the same biosynthetic pathway in *F. vesiculosus*. Initially, we were able to characterise the sugar-headgroup as *β*-galactopyranose based on the coupling constant values of H-1‴/H-2‴ (*J* = 7.2 Hz), H-2‴/H-3‴ (*J* = 9.3 Hz) and H3‴/H4‴ (*J* = 3.5 Hz) as well as NOE correlations observed from H-1‴ to H-3‴ and H-5‴. HMBC correlations from the anomeric proton H-1‴ (*δ*_H_ 4.28) to C-3 (*δ*_C_ 62.8) showed that the sugar unit was attached at the C-3 position of the glycerol moiety. Furthermore, the glycerol methylene protons H_2_-1 (*δ*_H_ 4.40, 4.21) correlated on the HMBC spectrum with C-1′ (*δ*_C_ 173.5) of the C20:5 chain, while the methylene protons at H-3″ (*δ*_H_ 1.65) correlated with the carbonyl carbon C-1″ (*δ*_C_ 173.3) of the second acyl chain (C18:4) linked at C-2; thus, confirming the regiochemical distribution of the two acyl chains as proposed via MS/MS fragment analysis. HMBC correlations from the methyl protons H_3_-20′ to C-18′ and from H-18″ to C-16″, revealed that both chains were derived from omega-3 fatty acids where the first double bond is three positions away from the lipid tail end [20]. In olefinic fatty acids, CH_2_ carbons adjacent to olefinic carbons are fixed and have characteristic ^13^C chemical shifts indicating their configuration [21,22]. Resonances around *δ*_C_ 25–28 are characteristic of an adjacent *cis*-double bond while more deshielded signals between *δ*_C_ 32–35 are of CH_2_ carbons adjacent to the *trans*-double bond [21,22]. Thus, the ^13^C NMR data for **1** were indicative of a *cis-*configuration (*Z* geometry) of the double bonds as chemical shifts of C-4′, C-7′, C-10′, C-13′, C-16′ and C-5″, C-8″, C-11″, C-14″ of both acyl chains were between *δ*_C_ 25.3–27.0. The double bond geometry and positions are also consistent with other macroalgal MGDGs [23,24]. The absence of NOE correlations from H-1‴ (*δ*_H_ 4.28) to H-2 (*δ*_H_ 5.31) suggested an *S*-configuration at C-2 position of the glycerol, which is consistent with the literature [24,25]. In nature, galactose mostly occurs in D-configuration [26]; hence, the structure of **1** was identified as (2*S*)-1-*O*-(5*Z*,8*Z*,11*Z*,14*Z*,17*Z*-eicosapentaenoyl)-2-*O*-(6*Z*,9*Z*,12*Z*,15*Z*-octadecatetranoyl)-3-*β*-d-galactopyranosyl-*sn-*glycerol-MGDG (20:5/18:4). This compound has been isolated from a Russian *F. evanescens* specimen [23].

Compounds **2**–**6** were isolated from the combined fractions H39–H42 and H45–H47. The NMR (Table S3) and the [α]_D_ data of the compounds **2**–**6** were almost identical to those of 1, with the only differences being in the chain lengths and number of double bonds in the fatty acyl portions of the molecules (Figure 2). On the basis of HR-MS/MS data (Figures S8, S10, S12, S14 and S16) and comparison to the reported NMR data [24,27,28], galactolipids **2**–**6** were identified as (2*S*)-1,2-*bis*-*O*-(6*Z*,9*Z*,12*Z*,15*Z*-octadecatetranoyl)-3-*β*-d-galactopyranosyl-*sn-*glycerol (**2**), (2*S*)-1-*O*-(9*Z*,12*Z*,15*Z*-octadecatrienoyl)-2-*O*-(6*Z*,9*Z*,12*Z*,15*Z*-octadecatetranoyl)-3-*β*-d-galactopyranosyl-*sn-*glycerol (**3**), (2*S*)-1,2-*bis*-*O*-(9*Z*,12*Z*,15*Z*-octadeca-trienoyl)-3-*β*-d-galactopyranosyl-*sn-*glycerol (**4**), (2*S*)-1-*O*-(5*Z*,8*Z*,11*Z*,14*Z*, 17*Z*-eicosapentaenoyl)-2-*O*-(9*Z*,12*Z*,15*Z*-octadecatrienoyl)-3-*β*-d-galactopyranosyl-*sn-*glycerol (**5**) and (2*S*)-1-*O*-(8*Z*,11*Z*,14*Z*,17*Z*-eicostetraenoyl)-2-*O*-(6*Z*,9*Z*,12*Z*,15*Z*-octadecatetranoyl)-3-*β*-d-galactopyranosyl-s*n*-glycerol (**6**).

Compound **7** was isolated from the bioactive *n*-hexane fractions H45–H47. Its molecular formula C_53_H_84_O_15_Na (*m/z* 983.5672 [M + Na]^+^) indicated the presence of an additional sugar unit. ^1^H NMR did not provide clear resonances for **7** due to broad signal overlap (Figure S18); thus, in-depth MS fragmentation pattern was used to identify the compound. The HR-MS/MS spectrum of the [M + Na]^+^ ion of **7** (Figure S19) contained fragment ions at *m/z* 821.5143 arising from the neutral loss of a galactose residue (Figure 2). Ions at *m/z* 705.3464 and *m/z* 681.3453 originated from the loss of C18:3 and C20:5 acyl substituents, respectively. The higher abundance of the latter, *m/z* 681.3453, indicated the C20:5 acyl chain to be at *sn*-1, while the C18:3 chain was located at *sn-*2 [19]. Fragment ions at *m/z* 543.2929 and *m/z* 519.2926 stemmed from the loss of a sugar unit plus the C18:3 and C20:5 acyl chains, respectively; *m/z* 405.1367 from the loss of both acyl chains together and *m/z* 347.0948 from the loss of digalactosyl moiety (Figure 2 and Figure S19). On the basis of this data, **7** was identified as digalactosyldiacylglycerol/DGDG (20:5/18:3).

All seven galactolipids were isolated in form of colourless oils that were best soluble in a CHCl_3_/MeOH/H_2_O (4:1:0.1) mixture. Compounds **1**–**5** and **7** have previously been isolated from various brown algae and microalgae [23,24,27]. Compound **6** has been detected in profiling studies on *F. vesiculosus* [29], however, to our knowledge this is the first time that compound **6** was isolated in a pure state. Here, we provide the first report of its HRMS/MS profile and ^1^H NMR data.

Next, we evaluated the antimicrobial activity of compounds **1**–**7**. The bioactive *n*-hexane fractions from which the galactolipids **1**–**7** originated potently inhibited MRSA between 65–85% at 100 µg/mL. However, as shown in Table 2, the purified galactolipids were inactive against MRSA at the same test concentration (100 µg/mL). Therefore, we included a second *S. aureus* strain (DSM346) for testing, which has not developed resistance against antibiotics. Compounds **1**–**4** did not show any antimicrobial activity against *S. aureus*, whereas the larger molecular weight compounds (**5**–**7**) exhibited moderate antimicrobial activity (Table 2). The MGDGs **5** and **6**, both *m/z* 821 [M + Na]^+^ and only differed by the number of double bonds in their acyl chains, had IC_50_ values of 96 and 123 µg/mL, respectively. The DGDG (**7**) was the most active compound that inhibited the drug-sensitive *S. aureus* strain with an IC_50_ value of 87 µg/mL.

### 2.3. Bioactive Molecular Netwoking of the F. vesiculosus n-BuOH Subextract

Out of the 29 fractions obtained by C18 HPLC from the *F. vesiculosus n*-BuOH subextract, eight fractions (B12, B14–19, B21) showed anti-MRSA inhibition above 70% and another eight fractions (B1-2, B11, B13, B20, B22, B28–29) above 50% at a test concentration of 100 µg/mL (Figure S1). Therefore, we repeated the bioassay also at a test concentration of 50 µg/mL and used these data to calculate the bioactivity scores in order to focus on the compounds with the highest activities. This revealed that the most MRSA-active compounds were present in fractions B14–17 with inhibition levels of 57–100%.

**Figure 3:**
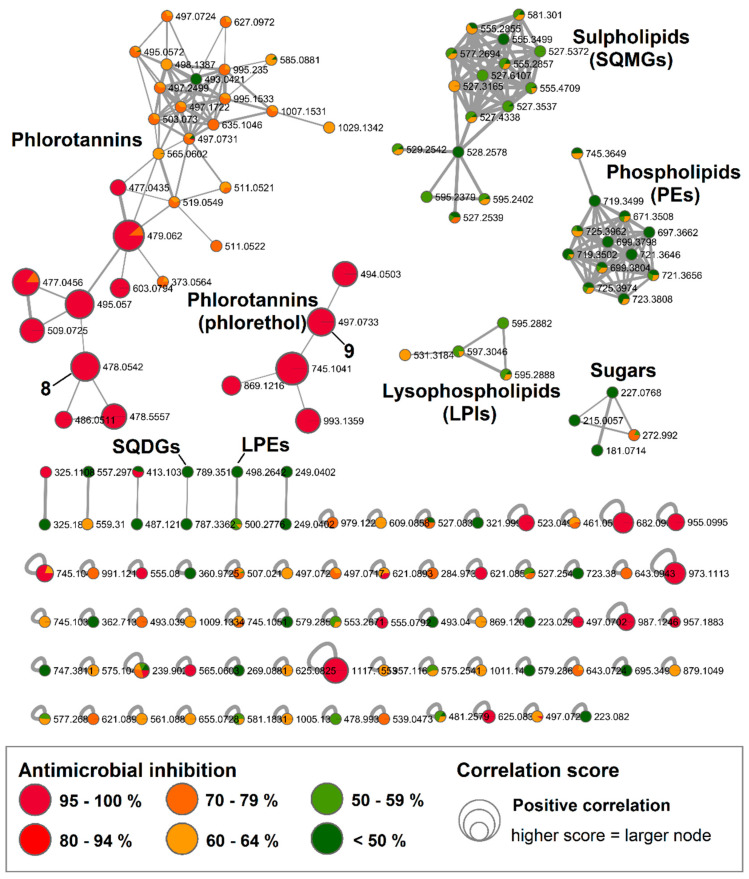
Bioactive molecular network of the *F. vesiculosus n*-BuOH subextract displaying compounds, in particular phlorotannins, with high bioactivity correlation scores. Nodes are coloured according to their inhibitory potency (%) against MRSA at 100 µg/mL concentration and node size reflects the bioactivity scores of the ions. Nodes of isolated compounds **8** and **9** are annotated in the network. (Data recorded in negative ionization mode).

UPLC-QToF-MS/MS data were acquired for all 29 *n*-BuOH fractions in negative mode to obtain the BMN, which consisted of 144 nodes and six distinct clusters (Figure 3). However, 62 ions were singletons and did not cluster with any other ion based on the MS/MS fragments, even after manipulation of network parameters such as “fragment ion mass tolerance”, number of “minimum matched fragment ions” and the “minimum cosine score”. Through the standard FBMN workflow, several clusters could be linked to known compound classes (Table S2 and Figure S20). This included sugars, either galacitol or mannitol, that are indiscernible by MS as both have the same molecular weight and similar MS fragmentation patterns (Figure S21). Molecular clusters for several types of polar lipids such as phospolipids, sulpholipids and lysophospolipids were also recognized in the *n*-BuOH subextract (Figure 3 and Figure S20). Based on the *F. vesiculosus* lipidomics data reported by da Costa et al. [29], we were able to manually annotate two more clusters to phospholipids: phosphatidylglycerols (PEs) and lysophosphatidylinositols (LPIs). Furthermore, we identified a large molecular family belonging to the sulfoquinovosyl monoglycerols (SQMGs) and two connected nodes belonging to sulfoquinovosyl diglycerol (SQDGs). Additionally observed was the lysophosphatidylethanolamine (LPE) 1-(5*Z*,8*Z*,11*Z*,14*Z*-eicosatetraenoyl)-*sn*-glycero-3-phospho-ethanolamine (*m/z* 500.2776 [M − H]^−^), annotated through GNPS (Figure S22).

The largest molecular family in the *n*-BuOH subextract was, however, the phlorotannins. Two distinct clusters and several single nodes featured specific phlorotannin fragmentation patterns. Phlorotannins are polymers of phloroglucinol that can reach high molecular weights. Depending on their linkage they are classified as fucols (linked through C-C bonds), phlorethols (linked with C-O bonds), fuhalols (linked with C-O bonds with an extra hydroxyl group on one unit) or eckol (with dioxane linkage) [30]. Due to the ether linkage they possess, phlorethols more readily produce MS/MS fragments [30]. In BMN, the smaller of the two phlorotannin clusters showed very high bioactivity scores and its ions were putatively annotated as phlorethols, since the MS/MS spectra showed fragment ions that represented the loss of one or more phloroglucinol moieties indicative by a loss of −124/126 Da or *O*-phloroglucinol moieties (−140/142 Da). On this basis, and by comparison to data reported in the literature, we could tentatively annotate two phlorethol-type ions, *m/z* 497.0733 [M − H]^−^ as fucodiphloroethol [9] and *m/z* 993.1359 [M − H]^−^ as pentafucodiphlorethol [31] (Table S2). In the large cluster, fucofurodiphlorethol (*m/z* 479.0607 [M − H]^−^) [31] and triphlorethol *(m/z* 373.0564 [M − H]^−^) [32] could be identified. The further three phlorotannins were dereplicated from single nodes: tetrafucol (*m/z* 497.0717 [M − H]^−^) [33], trifucophlorethol (*m/z* 621.0890 [M − H]^−^) [30] and hexafucol (*m/z* 745.1033 [M − H]^−^) [33] (Table S2). LC-MS/MS is a very powerful tool in the identification of phlorotannins and helps assigning the phlorotannin type, however the correct assignment of structures remains very challenging, since the exact position of the linkages between the individual phloroglucinol units cannot be determined, thus the annotated compounds (Figure S20) may occur as different phlorotannin isomers.

**Figure 4:**
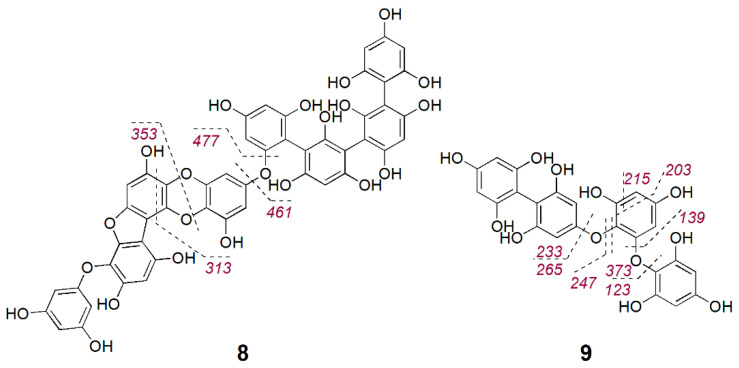
Tentative structures of phlorotannins (**8**–**9**) isolated from Baltic *F. vesiculosus.*

The larger phlorotannin cluster consisted of several putatively bioactive nodes in the range of *m/z* 477 to 603 [M − H]^−^ (Figure 3); however, a close inspection of the MS spectra revealed that the *m/z* 478.0542 [M − 2H]^2−^ (bioactivity score: *r* = 0.66, *p* > 0.05) was actually the doubly charge ion of the compound at *m/z* 957.1215 [M − H]^−^ (Figure S23). The molecular weight of this compound corresponded to the molecular formula C_48_H_30_O_22_, which did not return any hits in the databases used in this study. Therefore, we gave priority for the rapid and targeted isolation of this phlorotannin (**8**, 1.2 mg) as a red-brownish solid from the bioactive fraction B15. Initially, we acquired the NMR spectra for **8** in DMSO-*d_6_* that showed aromatic proton signals typical of phenolic compounds (Figures S24–S27). The ^1^H NMR spectrum (Figure S24) indicated that **8** is highly aromatic and symmetrical. Resonances for seven *meta*-coupling aromatic protons were observed at *δ*_H_ 5.85 (d, *J* = 2.3 Hz), *δ*_H_ 5.83 (d, *J* = 2.3 Hz), *δ*_H_ 5.77 (d, *J* = 2.1 Hz), *δ*_H_ 5.78 (d, *J* = 2.3 Hz), *δ*_H_ 5.71 (d, *J* = 2.1 Hz), *δ*_H_ 5.70 (d, *J* = 2.1 Hz), *δ*_H_ 5.65 (d, *J* = 2.1 Hz) (Table 3). The observed HSQC and HMBC correlations allowed assignment of a number of ^13^C signals around *δ*_C_ 94 and around *δ*_C_ 157 (Table 3), confirming the polyphenolic nature of **8**. However, signals in the 2D spectra appeared to be broadened and potentially missing, thus linkages of the different phloroglucinol units could not be elucidated based on the NMR data. Yotsu-Yamashita et al. [34] observed that proton signals of phlorotannins were best resolved in methanol-*d*_4_, thus we reacquired ^1^H NMR data in methanol-*d*_4_. This attempt, however, did not provide a better resolution but caused hydrogen-deuterium exchange, which could clearly be observed in the isotopic pattern of HR-MS spectrum that was acquired following NMR measurements (Figure S28). We attempted to reverse the hydrogen–deuterium exchange by drying and shaking the compound in non-deuterated MeOH. Unfortunately, the subsequent HPLC-ELSD and UPLC-MS analyses revealed that by this time the compound had degraded. The stability is a known issue for phlorotannins [35,36], peracetylation prior to their isolation has been suggested to hinder oxidation of phlorotannins and hydrogen–deuterium exchange [35]. Herein, due to the instability problem, we could not determine the full chemical structure or the antimicrobial activity of **8**. Based on the molecular weight and the thereof predicted molecular formula, **8** consists of eight phloroglucinol subunits. MS/MS fragment data of **8** showed an ion at *m/z* 477.0459 [M − H]^−^ potentially indicating the loss of three phloroglucinol moieties plus resorcinol (−3 × 124–108 Da) and another ion at *m/z* 461.0498 [M − H]^−^ due to the loss of four phloroglucinol units (Figure 4 and Figure S23). Considering the molecular formula of **8**, it is highly plausible that the remaining four phloroglucinol unites are linked via a dioxane and a furane group. Thus, the chemical structure of the new compound **8** was tentatively assigned as a phlorofucofuroeckol, as shown in Figure 4.

During our targeted isolation efforts, we also isolated another phlorotannin type compound, **9**, with a pseudomolecular ion at *m/z* 497.0733 [M − H]^−^ and molecular formula C_24_H_18_O_12_, which was identified as a fucophlorethol based on characteristic MS/MS fragmentation (Figure 4 and Figure S29). The fragment ion *m/z* 265.0803 [M − H]^−^ indicated the loss of one phloroglucinol moiety plus resorcinol (−124–108 Da), *m/z* 247.0695 [M − H]^−^ originated from the loss of two phloroglucinol moieties, *m/z* 233.0898 [M − H]^−^ from the loss of a phloroglucinol moiety and an *O*-phloroglucinol (−124–140 Da), *m/z* 139.0454 [M − H]^−^ from the residue *O*-phloroglucinol, and *m/z* 123.0496 [M − H]^−^ from the residue phloroglucinol. Several other fragment ions that originated from further cross-ring cleavages were also identified (Figure 4 and Figure S29). This structure agrees with a commonly reported fucodiphlorethol from *F. vesiculosus* in the literature [9,37]. Further phlorotannins with high bioactivity scores in Figure 3 only occurred in very minute amounts and could not be isolated in sufficient amounts in this study.

## 3. Discussion

Untargeted MS-based metabolomics complements many research areas; however, data interpretation still remains a challenging task. Undoubtedly, the GNPS molecular networking approach has advanced the dereplication process in natural product research and the annotation of complex mixtures. The recently introduced concept bioactive molecular networking (BMN) [13] links mass spectrometric features with biological activity. This high-throughput approach assists standard bioactivity-guided fractionation and other chemometric approaches (e.g., PLS or S-plots [38]) by highlighting new potential bioactive target molecules in a molecular network prior to costly and time-consuming isolation efforts. This methodology has been successfully implemented to isolate antiviral plant metabolites that had been missed in a former bioassay-guided fractionation approach [13]. Recently, we employed this technique for the targeted isolation of new anticancer decalinoyl-spirotetramic acid derivatives, pyrenosetins A–C, from the marine fungus *Pyrenochaetopsis* sp. associated with the thallus of Baltic *F. vesiculosus* [39]. In our year-long study of monthly monitoring of the metabolome and bioactivity profile of Baltic *F. vesiculosus*, the organic extracts of the seaweed displayed consistent antimicrobial activity against drug resistant human pathogens, particularly MRSA in all months [8], whereas untargeted UPLC-QToF-MS/MS analysis has pointed to a breadth of yet unidentified compounds in *F. vesiculosus* extracts [5]. Therefore, we set out to implement the BMN approach in order to profile the antimicrobial constituents of the Baltic *F. vesiculosus* and investigate their structure activity relationships.

In depth in silico and manual dereplication revealed that the *F. vesiculosus n*-hexane subextract was highly enriched in galactolipids; in particular, MGDGs, DGDGS, and DGMGs. This compound class was not observed in our previous detailed *F. vesiculosus* metabolome studies, which were performed on crude MeOH and DCM extracts [5,8]. In this study, we were able to detect these compounds after a rapid liquid–liquid partitioning of the methanol extract, followed by further fractionation. This highlights the power of the simple fractionation steps to reveal compounds with lower abundances that may be masked and missed in crude mixture analyses. The detected galactolipids in this study are known to be major constituents of photosynthetic membranes [40], and thus are commonly found in plants and algae, including *Fucus* sp. [29,41,42]. In photosynthetic membranes, MGDGs are of conical shape and are non-bilayer forming, while DGDGs can form bilayers and photosynthetic membranes are comprised of a combination of the two [17]. Besides their important role in stabilising membranes, galactolipids have been shown to exhibit a range of ecological and pharmacologically relevant activities, such as antiherbivore [43], antiinflammatory [44], antibacterial [45,46], antiviral [47] and antitumour [48,49]. A low natural abundance and the purification of single galactolipids have both been claimed as major challenges in galactolipid research [50]. Here, we isolated a broad range of six different MGDGs (**1**–**6**) and one DGDG (**7**) from Baltic *F. vesiculosus* to assess their antimicrobial structure–activity relationship. Overall, the antimicrobial potency of all tested galactolipids was low and the MRSA strain showed resistance against the tested compounds. In bioassays against the drug-sensitive *S. aureus* strain (DSM346), **1**–**4** showed also low inhibition rates (<50%) even at a higher test concentration (200 µg/mL). Generally, we observed that only compounds with a longer C20 acyl substitution showed inhibition against *S. aureus*. This is similar to the conclusion drawn by Colombo et al. [51], who studied the antitumor activity of different MGMGs and reported that the length of the acyl chain linked to the glycerol moiety was more important for antitumor activity than the position of the ester function or the nature of the sugar. Interestingly, compounds **1**, **5** and **6** shared a polyunsaturated eicosanoic acid substitution at *sn-*1 and a polyunsaturated octadecanoic acid at *sn*-2, but only 5 and 6 showed antimicrobial activity with IC_50_ values of 96 and 123 µg/mL, respectively. These three compounds only differed in the number of double bonds. This suggested that the degree of unsaturation played a positive role on antimicrobial activity, where the lower cumulative number of double bonds, in this case eight double bonds in MGDG (20:5/18:3) (**5**) and MGDG (20:4/18:4) (**6**) versus nine double bonds in MGDG (20:5/18:4) (**1**), resulted in higher IC_50_ values in our assay. Furthermore, we observed that the addition of a second galactose unit to the headgroup in the isolated DGDG (**7**) showed slightly enhanced activity against *S. aureus* (DSM346), thereby indicating that for antimicrobial activity the number of the sugar units plays a considerable role.

The bioactive fractions from which the galactolipids were derived were comprised of a mixture of galactolipids and initially showed up to 85% inhibition of MRSA at 100 µg/mL. This may indicate an additive or synergistic effect of the galactolipids. Notably, the bioactivity scores for the *n*-hexane fractions were significantly lower (*r* < 0.37) than the scores for the phlorotannins (*r* < 0.80); therefore, the observed low bioactivities were not entirely unexpected from a statistical point of view. However, compounds **1** and **4** showed higher statistical correlations with antimicrobial activity than the moderately active compounds **5**–**7**. These “false positive” results in the BMN may occur when compounds coelute and covary in a concentration shift in the same manner as a truly active compound. All isolated glycolipids are structurally very similar, and hence are very difficult to purify by chromatographic techniques. For instance, compounds **1**, **5** and **6** all coeluted together in the active fractions H40–H42 and also covaried in the very similar abundance patterns. In all these fractions, **1** was the most abundant and received a higher statistical correlation score compared to **5** and **6**. This phenomenon was also reported in a detailed study by Caesar et al. [52] that found—especially in low concentration data sets—that higher abundant ions were more prone to overfitting and may lead to false biological activity interpretations. The BMN approach readily pointed to potential classes of bioactive target compounds, i.e., galactolipids and phlorotannins; however, the actual bioactivity of the compounds was not accurately reflected in the bioactivity scores of the BMN. The modern MS technology is a favoured analytical tool in metabolomics due to its high sensitivity and ability to detect a broad range of different metabolites. However, when the analytical approach is more sensitive than the employed assay for bioactivity determination, it is highly possible to miss highly bioactive compounds with minute amounts [52]. This, however, is not only a limitation to BMN but to any bioassay-guided fractionation approach and shows the importance of isolation and testing of the pure compounds to confirm the predicted activities.

Fucoxanthin, which we also detected in the *n*-hexane subextract, is a photosynthetic pigment specific to brown algae and diatoms. A number of biological activities, such as antimicrobial, antioxidant and anticancer activities have been attributed to fucoxanthin [10,53]. In our BMN, antimicrobial activity did not correlate with fractions containing this carotenoid. Karpiński and Adamczak [54] reported the antibacterial activity of fucoxanthin with an MIC of 62.5 µg/mL against the human pathogen *Streptococcus agalactiae*. All other tested bacterial strains, including *S. aureus*, were only inhibited at MICs > 125 µg/mL, hence being in accordance with the predicted bioactivity scores for fucoxanthin in our BMN.

The *n*-BuOH subextract was rich in several classes of polar lipids but the most predominant constituents were phlorotannins, many of which were found in highly bioactive fractions. Phlorotannins are specific to brown algae and known to be present in physodes, membrane vesicles of the cytoplasm [55]. From the physodes they are released and incorporated into the cell walls of brown algae where they absorb harmful UV radiation. They give the algae their brown colouration [55], reportedly strengthen the algal cell walls [56] and play a role in wound healing [57]. However, a large fraction of phlorotannins in brown algae remain soluble in the physodes and can be released to the environment, hence are thought to have multiple secondary ecological functions [58], such as antifouling and antiherbivore roles [59]. Generally, a higher degree of phloroglucinol polymerisation resulted in higher biological activity [60]. Reported antimicrobial activities (IC_50_) against *S. aureus*, MRSA and a number of other human pathogens of seaweed-derived phlorotannins ranged from 32 to 250 µg/mL [61]. The higher antimicrobial activity levels of reported phlorotannins compared to galactolipids appear to be reflected through our BMN approach that showed higher bioactivity scores for phlorotannins than for galactolipids. Further isolations and biological activity testing of phlorotannins are required to confirm this suggestion.

Although phlorotannins are biosynthetic end products, they can further degrade via demethylation, hydration, or oxidation [58]. This inherent instability of phlorotannins has made their extraction and isolation very challenging and often pre-derivatisation steps are required for successful purification and characterisation [36]. The aromatic protons between two phenolic groups are prone to hydrogen-deuterium exchange with deuterated NMR solvents (e.g., CD_3_OD) that can lead to decreased or fully exchanged peaks in NMR spectra [35]. Acetylation of the hydroxyls prevents keto-enol tautomerization and thus supresses oxidisation [35]. However, the antimicrobial activity of phlorotannins has been associated with the ability of their free phenolic groups to bind to amine groups of bacterial proteins [62], thus acetylation could result in reduced or even loss of activity. The reactivity of phlorotannins can differ significantly even between related species; thus established procedures in terms of temperature, solvents and handling times for one organism might cause degradation of phlorotannins in another [35]. Consequently, only a few successful studies have fully characterised phlorotannins (e.g., [63,64,65,66,67,68,69,70]). NMR spectroscopy of phlorotannins can be very challenging due to the high structural similarity and is generally complemented with tandem mass spectrometry that provides useful information about the nature of the linkages between the individual phloroglucinol units [35]. Here, due to the instability of compound **8**, we mostly based our chemical characterisation on MS/MS fragmentation pattern and identified a putatively new and bioactive phlorofucofuroeckol (**8**). In future, we will revisit an optimised and targeted isolation of the phlorotannins that showed high bioactivity scores.

Algae have an important place in Asian cuisine and traditional medicine, but also in modern biotechnology. This study showed that the *F. vesiculosus* metabolome is comprised of many valuable constituents. Particularly in the *n*-hexane extract, we detected vitamins (α-tocopherol), and a great variety of lipids including many omega-3 fatty acids (e.g., in MGDGs and DGDGs) of high nutritional value as they promote cardiovascular health. Thus, this supports the use of *F. vesiculosus* in functional foods [29]. Despite the moderate antibacterial activity of the pure compounds, the *n-*hexane subextracts and fractions showed higher inhibition. This may suggest potential application areas for the more easily produced lipophilic extracts, e.g., in antibacterial cosmetic products. Phlorotannins, on the contrary—even though they have been reported to possess favourable antimicrobial as well as antioxidant properties—have limited use in functional foods as they easily degrade [36], but similarly to the galactolipids, they might remain more active and stable in the form of fractions.

In conclusion, we herein demonstrated that galactolipids and phlorotannins, constituents of membranes and cell walls, are responsible for the antimicrobial activity of *F. vesiculosus*. These metabolites are of high ecological relevance to the seaweed as they can regulate biofilm formation on the algal surface and thus provide a competitive advantage as these metabolites protect the seaweed from pathogens and excessive epibiosis [62]. The findings of this study offer further evidence that not only one compound class, but several, contribute to the observed antimicrobial activity of *F. vesiculosus*. BMN revealed to be a promising approach especially for lipidomic studies of algae as different lipid classes readily formed different clusters and were easily annotated in the molecular networks. Overall, this study has shown that the BMN approach can be a useful guide to focus isolation efforts on specific bioactive targets, but its potential limitations, e.g., over-representing high abundance ions have to be considered and addressed in future studies. The chemical isolation of the putatively bioactive compounds will reveal the true potency and bioactivity profiles of the metabolites.

## 4. Materials and Methods

### 4.1. General Procedures

Metabolomic data were acquired on an Acquity UPLC I-Class System coupled to a Xevo G2-XS QTof Mass Spectrometer (Waters^®^, Milford, MA, USA) with an Acquity UPLC HSS T3 column (High Strength Silica C18, 1.8 µm, 2.1 × 100 mm, Waters). NMR spectra were recorded on a Bruker AV 600 or a Bruker Avance III 400 NMR spectrometer (Bruker^®^, Billerica, MA, USA). Optical rotations were measured on Jasco P-2000 polarimeter (Jasco, Pfungstadt, Germany). Normal phase flash chromatography was performed with silica (200–400 mesh, Bio-Rad Laboratories GmbH, Feldkirchen, Germany). Preparative HPLC was performed on a Gemini-NX C18 column (50 × 100 mm, Phenomenex, Torrance, CA, USA) attached to a LaPrep system consisting of a P110 pump (VWR International, Allison Park, PA, USA) with a Dynamic Mixing Chamber (Knauer, Germany), P311 UV/VIS and Labocol Vario-2000 Fraction Collector (Labomatic, Switzerland). HPLC separations were achieved on a VWR Hitachi Chromaster HPLC system (VWR International, Allison Park, PA, USA) consisting of a 5430 diode array detector (VWR International, Allison Park, PA, USA), a 5310 column oven, a 5260 autosampler and a 5110 pump combined in parallel with a VWR Evaporative Light Scattering Detector (ELSD 90). Compound purifications were performed on semipreparative C18 monolithic column (Onyx, 100 × 10 mm, Phenomenex, Torrance, CA, USA) and Synergi^TM^ polar-RP 80 Å LC column (250 × 10 mm, Phenomenex, Torrance, CA, USA). UPLC solvents were purchased from Biosolve (Valkenswaard, Netherlands). The water used was MilliQ-water filtered in-house on Arium^®^ Water Purification Systems (Sartorius, Göttingen, Germany). *n*-Hexane, dichloromethane, methanol and acetonitrile were purchased from AppliChem GmbH (Hannover, Germany). *n*-Butanol and NMR solvents (CDCl_3_, CD_3_OD and DMSO-*d*_6_) were from Roth GmbH (Karlsruhe, Germany).

### 4.2. Biological Material

*Fucus vesiculosus* material (1 kg) was collected from a littoral patch at Bülk, outer Kiel Fjord, in the Baltic Sea (54°27′15.3 N, 10°11′56.1 E) on 12 July 2018. The algal material was placed in zip-lock bags containing seawater, which were transported back to the laboratory on ice. In the laboratory, the *F. vesiculosus* material was rinsed with deionised water to remove epibionts and excess salt. Subsequently, the algal material was freeze-dried and pulverised.

### 4.3. Extraction, Fractionation and Isolation

A total of 873 g of dried and pulverised *F. vesiculosus* material was divided into four parts that were each placed in a 2 L Erlenmeyer. A total of 800 mL of MeOH was added to each flask and stirred overnight (2000 rpm, IKA^®^ RH basic 2, Staufen, Germany). The extraction was repeated 3 times and the MeOH extract was decanted through a cellulose filter (210 mm) and evaporated *in vacuo* to yield a total of 160 g of methanolic extract. A modified Kupchan liquid–liquid partition scheme was performed [14]; 62 g of the crude extract was suspended in 10% aqueous MeOH (800 mL) and partitioned against *n*-hexane (4 × 800 mL). The water content was then increased to 30% and the aqueous MeOH phase was successively partitioned against dichloromethane (3 × 1 L). Due to the high salt content in the aqueous MeOH phase, it was also partitioned against *n*-BuOH (3 × 800 mL). The solvents were removed to yield a nonpolar *n*-hexane subextract (13 g), a middle polarity DCM subextract (7 g), and the polar *n*-BuOH (7 g) and an aqueous phase (34 g). Only the bioactive *n*-hexane and *n*-BuOH subextracts were analysed further.

For bioactivity mapping, 10 g of the nonpolar *n*-hexane subextract was fractionated by silica flash chromatography (200 g SiO_2_). The subextract was flushed with 100% *n*-hexane and then eluted with a stepwise gradient (5%) of EtOAc and finally flushed with 100% EtOAc. A total of 40 fractions (H8–H48, each 200 mL) were collected. The polar *n*-BuOH subextract (5 g) was dissolved in 50 mL of 50% aqueous MeOH. The extract was centrifuged (3000 rpm, 15 min) to precipitate poorly soluble polysaccharides, and preparative RP-HPLC was employed using a preparative Gemini-NX C18 column (50 × 100 mm, Phenomenex) to fractionate the supernatant in eight successive HPLC runs into 29 subfractions (B1–B29) with the following gradient: 0–5 min H_2_O:MeOH (99:1), 5–23 min linear from 1–100% MeOH, 23–30 min 100% MeOH (flow 30 mL/min).

The combined active fractions containing galactolipids H39–32 (430 mg) were subjected to semi-preparative RP–HPLC equipped with an Onyx (100 × 10 mm, Phenomenex) C18 monolithic column (gradient of H_2_O/MeCN with 0.1% FA from 20:80 to 10:90 in 30 min, flow 2.5 mL/min, column oven 40 °C) to yield compound **1** (1.0 mg, t_R_ 18 min), compound **3** (1.6 mg, t_R_ 20 min), compound **4** (1.3 mg, t_R_ 25.2 min), compound **5** (1.3 mg, t_R_ 22.8 min), and compound **6** (0.5 mg, t_R_ 24 min). Fractions H45–47 (238 mg) were combined and eluded with the same gradient as mentioned above to give compound **2** (0.4 mg, t_R_ 21 min) and compound **7** (0.9 mg, t_R_ 20 min) in a pure state.

The RP–HPLC chromatography equipped with using a Synergi^TM^ polar-RP 80 Å LC column (250 × 10 mm, Phenomenex) of the phlorotannin-containing bioactive fraction B15 (56 mg) (gradient of H_2_O/MeCN with 0.1% FA from 80:20, 0–2 min; 80:20 to 55:45, 2–15 min; 55:45, 15–18 min; 55:45 to 10:90, 18–25 min, flow 2.5 mL/min, column oven 30 °C) yielded compound **8** (1.2 mg, t_R_ 8 min) and **9** (0.1 mg, t_R_ 12 min).

(2*S*)-1-*O*-(5*Z*,8*Z*,11*Z*,14*Z*,17*Z*-eicosapentaenoyl)-2-*O*-(6*Z*,9*Z*,12*Z*,15*Z*-octadecatetranoyl)-3-*β*-d-galactopyranosyl-*sn-*glycerol/MGDG (20:5/18:4) (**1**): Colourless oil; [α]${}_{D}^{20}$ −12 (*c* 0.1, CHCl_3_); ^1^H NMR (CDCl_3_, 600 MHz) and ^13^C NMR (CDCl_3_, 150 MHz), Table 1; (+)-HR-ESIMS found *m*/*z* 819.5018 [M + Na]^+^ C_47_H_72_O_10_Na, calculated 819.5023; deposited in the GNPS spectral library, https://gnps.ucsd.edu/ProteoSAFe/gnpslibraryspectrum.jsp?SpectrumID=CCMSLIB00005723382#%7B%7D

(2*S*)-1,2-*bis*-*O*-(6*Z*,9*Z*,12*Z*,15*Z*-octadecatetranoyl)-3-*β*-d-galactopyranosyl-*sn-*glycerol/MGDG (18:4/18:4) (**2**): Colourless oil; [α]${}_{D}^{20}$ −12 (*c* 0.1, CHCl_3_); ^1^H NMR (CDCl_3_, 400 MHz), Table S3; (+)-HR-ESIMS found *m/z* 793.4849 [M + Na]^+^ C_45_H_70_O_10_Na, calculated for 793.4867; deposited in https://gnps.ucsd.edu/ProteoSAFe/gnpslibraryspectrum.jsp?SpectrumID=CCMSLIB00005723381#%7B%7D

(2*S*)-1-*O*-(9*Z*,12*Z*,15*Z*-octadecatrienoyl)-2-*O*-(6*Z*,9*Z*,12*Z*,15*Z*-octa-decatetranoyl)-3-*β*-d-galacto-pyranosyl-*sn-*glycerol/MGDG (18:3/18:4) (**3**): Colourless oil; [α]${}_{D}^{20}$ −18.3 (*c* 0.1, CHCl_3_); ^1^H NMR (CDCl_3_, 400 MHz), Table S3; (+)-HR-ESIMS found *m/z* 795.5009 [M + Na]^+^ C_45_H_72_O_10_Na, calculated for 795.5023; deposited in the GNPS spectral library, https://gnps.ucsd.edu/ProteoSAFe/gnpslibraryspectrum.jsp?SpectrumID=CCMSLIB00005723380#%7B%7D

(2*S*)-1,2-*bis*-*O*-(9*Z*,12*Z*,15*Z*-octadecatrienoyl)-3-*β*-d-galactopyranosyl-*sn-*glycerol/MGDG (18:3/18:3) (**4**): Colourless oil; [α]${}_{D}^{20}$ −13.8 (*c* 0.1, CHCl_3_); ^1^H NMR (CDCl_3_, 400 MHz), Table S3; (+)-HR-ESI-MS found *m/z* 797.5190 [M + Na]^+^ C_45_H_74_O_10_Na, calculated for 797.5180; deposited in the GNPS library, https://gnps.ucsd.edu/ProteoSAFe/gnpslibraryspectrum.jsp?SpectrumID=CCMSLIB00005723383#%7B%7D

(2*S*)-1-*O*-(5*Z*,8*Z*,11*Z*,14*Z*,17*Z*-eicosapentaenoyl)-2-*O*-(9*Z*,12*Z*,15*Z*-octadecatrienoyl)-3-*β*-d-galacto-pyranosyl-*sn-*glycerol/MGDG (20:5/18:3) (**5**): 0.5 mg, colourless oil; [α]${}_{D}^{20}$ −18.3 (*c* 0.1, CHCl_3_); ^1^H NMR (CDCl_3_, 400 MHz), Table S3; (+)-HR-ESIMS found *m/z* 821.5193 [M + Na]^+^ C_47_H_74_O_10_Na, calculated for 821.5180; deposited in the GNPS spectral library, https://gnps.ucsd.edu/ProteoSAFe/gnpslibraryspectrum.jsp?SpectrumID=CCMSLIB00005723384#%7B%7D

(2*S*)-1-*O*-(8*Z*,11*Z*,14*Z*,17*Z*-eicostetraenoyl)-2-*O*-(6*Z*,9*Z*,12*Z*,15*Z*-octadecatetranoyl)-3-*β*-d-galacto-pyranosyl-sn-glycerol/MGDG (20:4/18:4) (**6**): Colourless oil; [α]${}_{D}^{20}$ −14.2 (*c* 0.1, CHCl_3_); ^1^H NMR (CDCl_3_, 400 MHz), Table S3; (+)-HR-ESI-MS found *m/z* 821.5180 [M + Na]^+^ C_47_H_74_O_10_Na, calculated for 821.5180; deposited in the GNPS spectral library, https://gnps.ucsd.edu/ProteoSAFe/gnpslibraryspectrum.jsp?SpectrumID=CCMSLIB00005723385#%7B%7D

DGDG (20:5/18:3) (**7**): Colourless oil; [α]${}_{D}^{20}$ −12.5 (*c* 0.1, CHCl_3_); ^1^H NMR (CDCl_3_, 400 MHz), Figure S18; (+)-HR-ESIMS found *m/z* 983.5672 [M + Na]^+^ C_53_H_84_O_15_Na, calculated for 983.5678; deposited in https://gnps.ucsd.edu/ProteoSAFe/gnpslibraryspectrum.jsp?SpectrumID=CCMSLIB00003134754#%7B%7D

Compound **8**: Red-brown solid; partial NMR data (DMSO-*d*_6_, 600 MHz), Table 3 and Figures S24–S27. (−)-HR-ESIMS found *m/z* 957.1215 [M − H]^−^ C_48_H_29_O_22_, calculated for 957.1206.

Compound **9**: Red-brown solid; (−)-HR-ESIMS found *m/z* 497.0733 [M − H]^−^ C_24_H_17_O_12_, calculated for 497.0728.

### 4.4. LC-MS/MS-Based Bioactivity Molecular Networking

The *F. vesiculosus* fractions were analysed by an Acquity UPLC I-Class System coupled to a Xevo G2-XS QTof Mass Spectrometer (Waters^®^, Milford, MA, USA). Before injection, all samples were dissolved in MeOH and filtered through 0.2 µm PTFE syringe filters (Carl Roth, Karlsruhe, Germany). Samples were prepared at a concentration of 0.1 mg/mL and injected (1 µL) onto an Acquity UPLC HSS T3 column (High Strength Silica C18, 1.8 µm, 2.1 × 100 mm, Waters^®^) operating at 40 °C. A binary mobile phase system (A: 0.1% formic acid in water UPLC/MS grade and B: 0.1% formic acid in acetonitrile) was pumped at a flow rate of 0.6 mL/min. The following gradients were adapted: *n*-hexane subfractions—0–7 min linear 70% to 100% B; 11.5–12.5 min 100% B; *n*-BuOH subfractions—0–11.5 min linear from 1 to 100% B, 11.5–12.5 min 100% B. The last 2.5 min of all runs were used to recondition the column. Total run time was 15 min. An optimised data-dependent acquisition mode was used consisting of a full positive mode MS survey scan covering the mass range between 50–1600 Da at 0.1 s scans, followed by an MS/MS scan of the five most intense ions. For fragmentation, a collision energy ramp with low collision energy of 6–60 eV and high collision energy of 9–80 eV was applied. The spectrometer settings were: capillary voltage: 3.0 kV (positive mode) and 3.5 kV (negative mode), cone voltage: 30 V, source temperature: 150 °C, cone gas flow: 50 L/h, desolvation gas flow: 1200 L/h. Samples were run in triplicate to identify injection errors, then one consensus chromatogram was picked for further bioinformatic analysis.

The raw data files were converted to mzXML format using ProteoWizard and then imported to MZmine v2.37 for preprocessing. The mass detection was set to a noise level of 1000 for the MS1 level and 50 for MS2 levels. The chromatogram was built with ions showing a minimum time span of 0.01, minimum height of 2500 and *m/z* tolerance 0.01 (or 5 ppm). The chromatogram was deconvoluted with the baseline algorithm (minimum peak height 2500, peak duration 0.01–0.25, and baseline level 1000). Deisotoping of the chromatogram was achieved by the isotope peak grouper algorithm with *m/z* tolerance of 0.01 (or 5 ppm) and RT tolerance 0.1 min. All samples were combined in a peak list using the join aligner algorithm; the data were duplicate peak filtered and ions detected in the solvent blanks were removed from the mass list. Only data with MS2 scans were exported as *csv* and *mgf* files and uploaded to the GNPS platform for feature-based molecular networking analysis [12]. The data were filtered by removing all MS/MS fragment ions within +/− 17 Da of the precursor *m/z*. MS/MS spectra were window filtered by choosing only the top 6 fragment ions in the +/− 50 Da window throughout the spectrum. For molecular networking of the *n*-hexane subextract (https://gnps.ucsd.edu/ProteoSAFe/status.jsp?task=c3414fdee6a94ac7876c86b4539a8d36), the precursor ion mass tolerance was set to 0.02 Da and the MS/MS fragment ion tolerance to 0.02 Da. A molecular network was then created where edges were filtered to have a cosine score above 0.7 and more than 6 matched peaks. While for molecular networking of the *n*-BuOH subextract (https://gnps.ucsd.edu/ProteoSAFe/status.jsp?task=c38c712eb5464fcf88b404f0bd437c3f), the precursor ion mass tolerance was set to 0.02 Da and the MS/MS fragment ion tolerance to 0.5 Da. A molecular network was then created where edges were filtered to have a cosine score above 0.65 and more than 4 matched peaks. For both datasets the subsequent parameters were as follows: edges between two nodes were kept in the network if and only if each of the nodes appeared in each other’s respective top 10 most similar nodes. Finally, the maximum size of a molecular family was set to 100, and the lowest scoring edges were removed from molecular families until the molecular family size was below this threshold. The analogue search mode was used by searching against MS/MS spectra with a maximum difference of 100.0 in the precursor ion value. The library spectra were filtered in the same manner as the input data. All matches kept between network spectra and library spectra were required to have a score above 0.7 and at least 6 matched peaks. A publicly accessible *R* script available at (https://github.com/DorresteinLaboratory/Bioactive_Molecular_Networks/blob/master/Bioactive_Molecular_Networks_v1.1_MZmine2.r) was used to determine a bioactivity score for each ion in the samples. Samples were first scaled by normalizing the intensity of the TIC and then the Pearson correlation score (*r*) and its significance were calculated between the peak area of an ion and the bioactivity level. The outputted node attribute table was incorporated into the Cytoscape software to visualize the molecular network and to map out the bioactivity scores. The data used for the molecular networking analysis were deposited in the MassIVE Public GNPS database (http://massive.ucsd.edu) under access number MSV000085539.

### 4.5. UNPD in Silico MS/MS Database and Manual Dereplication

For annotation of the networks, the automated GNPS dereplication workflow in combination with the Universal Natural Product Database (UNPD)-ISDB workflow was employed [15]. Therefore, the raw UPLC-QToF-MS/MS data were submitted to GNPS (version 1.2.3.) (https://gnps.ucsd.edu/ProteoSAFe/status.jsp?task=59bc27b00c184d08a577697d2c3bb464# and https://gnps.ucsd.edu/ProteoSAFe/status.jsp?task=c871df6c9c0d4421b09607a0ea2d8e00). The data was filtered by removing all MS/MS peaks within +/− 17 Da of the precursor m/z. MS/MS spectra were window filtered by choosing only the top 6 peaks in the +/− 50 Da window throughout the spectrum. The data were then clustered with MS-Cluster with a parent mass tolerance of 0.02 Da and a MS/MS fragment ion tolerance of 0.02 Da to create consensus spectra. Further, consensus spectra that contained less than 2 spectra were discarded. A network was then created where edges were filtered to have a cosine score above 0.7 and more than 3 matched peaks. Further edges between two nodes were kept in the network if and only if each of the nodes appeared in each other’s respective top 10 most similar nodes. The spectra in the network were then searched against GNPS spectral libraries. The library spectra were filtered in the same manner as the input data. All matches kept between network spectra and library spectra were required to have a score above 0.7 and at least 3 matched peaks. Subsequently, the clustered data were downloaded. Using an UBUNTU virtual machine, the clustered spectra *mgf* file and molecular networking attribute *out* files were then searched against the UNPD-ISDB (available at http://oolonek.github.io/ISDB/) where the library search parameters were adjusted to TOLERANCE = 0.005, SCORE_THRESHOLD = 0.2 and TOP_K_RESULTS = 5. The in silico dereplication results were carefully check for their feasibility, proofing MS/MS fragment spectra and considering biological sources of reported hits.

Furthermore, the chromatograms were manually dereplicated by calculating putative molecular formulae in MassLynx v4.1 and searching them against the Dictionary of Natural Products (http://dnp.chemnetbase.com), MarinLit (http://pubs.rsc.org/marinlit/) and DEREP_NP [71] databases.

### 4.6. Antimicrobial Activity

Antimicrobial assays were performed using Methicillin-resistant *Staphylococcus aureus* DSM18827 and *Staphylococcus aureus* DSM346. Overnight cultures of the test organisms were prepared and diluted to an optical density (600 nm) of 0.01. To prepare the assay, the samples (20 mg/mL stock solution) were dissolved in medium and transferred into a 96-well microtiter plate and 200 µL of the diluted culture was added to each well. The inoculated microplates were incubated for 5 h at 37 °C and 200 rpm. To detect the inhibitory effect of the substances 10 µL of a resazurin solution (0.3 mg/mL phosphate-buffered saline) was added to the microplate, incubated again for 5 min and the fluorescence signal (560 nm/590 nm) was measured using the microplate reader (Tecan Infinite M200, Tecan, Crailsheim, Germany). For IC_50_ determination a dilution series was prepared and the IC_50_ value was calculated by Microsoft Excel as the concentration that show 50% inhibition of viability on the basis of a negative control (no compound). The resulting values were compared with the positive control (chloramphenicol) and the DMSO control on the same plate.

## Figures and Tables

**Table 1:** NMR data for compound **1** MGDG (20:5/18:4) (CDCl_3_, 600 MHz).

Position	*δ*_H_ Multiplicity (*J* in Hz)	*δ*_C_	HMBC	NOE
1	4.40 dd (12.1, 3.5)	62.4	C2, C1′	H2
	4.21 dd (12.1, 6.5)			
2	5.31 m	69.9	-	H1, H3
3	3.98 dd (12.1, 5.8) 3.75 (11.3, 6.3)	62.8	C1, C2, C3‴	H2, H1‴
1′	-	173.5	-	
2′	2.33 m	33.2	C1′, C3′	
3′	1.69 m	24.4	C1′, C5′, C2′	
4′	2.07 m	26.5	C2′, cluster1	
5′, 6′, 8′, 9′, 11′, 12′, 14′, 15′, 17′, 18′ (cluster1)	5.31–5.39 m	126.8–131.8	cluster2	
7′, 10′, 13′, 16′ (cluster2)	2.81 m	25.3–26.6	cluster1	
19′	2.08 m	20.3	C20′, cluster1	
20′	0.97 t (7.5)	14.0	C18′, C19′	
1″	-	173.3	-	
2″	2.35 m	33.8	C1″, C3″, C4″	
3″	1.65 m	24.5	C1″, C2″, C4″	
4″	1.38 m	28.7	C5″, cluster 1	
5″	2.07 m	27.0	cluster 1	
6″, 7″, 9″, 10″, 12″, 13″, 15″, 16″ (cluster1)	5.31–5.39 m	126.8–131.8	cluster2	
8″, 11″, 14″ (cluster2)	2.81 m	25.3–26.6	cluster1	
17″	2.07 m	20.3	C18′, cluster1	
18″	0.97 t (7.5)	14.0	C16″, C17″	
1‴	4.28 d (7.2)	103.7	C3	H3‴, H5‴, H1, H3
2‴	3.63 dd (9.3, 7.2)	71.4	C1‴, C3‴	H3
3‴	3.59 dd (9.3, 3.5)	73.2	C2‴	H1‴, H4‴
4‴	4.01 dd (3.5, 0.6)	69.3	C2‴, C3‴	H3‴, H5‴
5‴	3.55 m	74.3	-	H1‴, H4‴, H6‴
6‴	3.98 m 3.86 dd (11.4, 6.8)	62.8	-	H5‴

**Table 2:** Antimicrobial activity of galactolipids **1**–**7**. Positive control: Chloramphenicol.

Compound	MRSA	*S. aureus* (DSM346)	
	Inhibition Rate (%, 100 µg/mL)	Inhibition Rate (%, 200 µg/mL)	IC_50_ (µg/mL)
**1**	-	40	>200
**2**	-	49	>200
**3**	-	32	>200
**4**	-	49	>200
**5**	-	60	96 (±4)
**6**	-	62	123 (±8)
**7**	-	66	87 (±4)
Positive control	100	100	0.5 (±0.0)

**Table 3:** NMR data for compound **8** (DMSO-*d*_6_, 600 MHz).

*δ*_H_ Multiplicity (*J* in Hz)	*δ*_C_	HMBC H→C
5.65 d (2.1)	94.1	
5.70 d (2.1)	94.3	94.5; 157.7
5.71 d (2.1)	94.3	
5.77 d (2.1)	94.2	94.5; 157.5
5.78 d (2.3)	95.6	
5.83 d (2.3)	94.2	158.5
5.85 d (2.3)	94.2	
8.4 s	-	94.5; 157.7

## Supplementary Materials

The following are available online at https://www.mdpi.com/1660-3397/18/6/311/s1. MRSA inhibitory activity data of all subextracts and fractions; dereplication tables for the *n*-hexane and *n*-BuOH subextracts; spectral data for compounds **1**–**9**.
