# Supplementary material for: Bioactive Molecular Networking for Mapping the Antimicrobial Constituents of the Baltic Brown Alga Fucus vesiculosus
Source: Mar Drugs. 2020 Jun 13;18(6):311. doi: 10.3390/md18060311 (PMC7345172; doi:10.3390/md18060311)
Supplement: Supplementary file 1 [file marinedrugs-18-00311-s001.pdf]

Supplementary Information

Bioactive Molecular Networking for Mapping the Antimicrobial Constituents of the Baltic Brown Alga *Fucus vesiculosus*

Larissa Buedenbender [1], Francesca Anna Astone [1] and Deniz Tasdemir [1, 2,*]

[1] GEOMAR Centre for Marine Biotechnology (GEOMAR-Biotech), Research Unit Marine Natural Products Chemistry, GEOMAR Helmholtz Centre for Ocean Research Kiel, Am Kiel-Kanal 44, Kiel 24106, Germany; lbuedenbender@geomar.de; francesastone@gmail.com

[2] Faculty of Mathematics and Natural Sciences, Kiel University, Christian-Albrechts-Platz 4, Kiel 24118, Germany

* Correspondence: dtasdemir@geomar.de; Tel.: +49-431-600-4430

List of Tables

	Page
**Table S1.** Major ions and their putative annotations of compounds detected in the *F. vesiculosus* *n*-hexane subextract	2
**Table S2.** Major ions and their putative annotations of compounds detected in the *F. vesiculosus* *n*-BuOH subextract	5
**Table S3.** ^{1}H NMR (400 MHz) data of galactolipids **1** – **6** in $CDCl_3$ (δ in ppm)	7

List of Figures

**Figure S1.** MRSA inhibitory activity (% at 100 μg/mL) of A) *F. vesiculosus* crude and subextracts B) *n*-hexane subfractions C) *n*-BuOH subfractions	8
**Figure S2.** GNPS MS/MS mirror plot of experimental and library data of α-tocopherol	9
**Figure S3.** Chemical structures of annotated metabolites in the *F. vesiculosus* *n*-hexane extract	10
**Figure S4.** Structure and labelled MS/MS product ions observed in the ESI-MS/MS spectrum of **1**	11
**Figure S5.** ^{1}H NMR spectrum of **1** ($CDCl_3$, 600 MHz)	11
**Figure S6**. HMBC NMR spectrum of MGDG (20:5/18:4) (**1**) ($CDCl_3$, 600 MHz)	12
**Figure S7.** NOESY NMR spectrum of MGDG (20:5/18:4) (**1**) ($CDCl_3$, 600 MHz)	12
**Figure S8.** Structure and labelled MS/MS product ions observed in the ESI-MS/MS spectrum of **2**	13
**Figure S9.** ^{1}H NMR spectrum of **2** ($CDCl_3$, 400 MHz)	13
**Figure S10.** Structure and labelled MS/MS product ions observed in the ESI-MS/MS spectrum of **3**	14
**Figure S11**. ^{1}H NMR spectrum of **3** ($CDCl_3$, 400 MHz)	14
**Figure S12.** Structure and labelled MS/MS product ions observed in the ESI-MS/MS spectrum of **4**	15
**Figure S13.** ^{1}H NMR spectrum of **4** ($CDCl_3$, 400 MHz)	15
**Figure S14.** Structure and labelled MS/MS product ions observed in the ESI-MS/MS spectrum of **5**	16
**Figure S15**. ^{1}H NMR spectrum of **5** ($CDCl_3$, 400 MHz)	16
**Figure S16.** Structure and labelled MS/MS product ions observed in the ESI-MS/MS spectrum of **6**	17
**Figure S17.** ^{1}H NMR spectrum of **6** ($CDCl_3$, 400 MHz)	17
**Figure S18.** ^{1}H NMR spectrum of **7** ($CDCl_3$, 400 MHz)	18
**Figure S19.** Structure and labelled MS/MS product ions observed in the ESI-MS/MS spectrum of **7**	18
**Figure S20.** Chemical structures of annotated metabolites in the *F. vesiculosus* *n*-BuOH extract	19
**Figure 21.** GNPS MS/MS mirror plot of experimental and library data of mannitol	20
**Figure 22.** GNPS MS/MS mirror plot of experimental and library data of 1-(5Z,8Z,11Z,14Z-eicosatetraenoyl)-*sn*-glycero-3-phospho-ethanolamine	20
**Figure S23.** HR-ESI-MS and MS/MS spectra of compound **8**	21
**Figure S24**. ^{1}H NMR spectrum of compound **8** (DMSO-d_6, 600 MHz)	21
**Figure S25**. COSY spectrum of compound **8** (DMSO-d_6, 600 MHz)	22
**Figure S26.** HSQC spectrum of compound **8** (DMSO-d_6, 600 MHz)	22
**Figure S27.** HMBC spectrum of compound **8** (DMSO-d_6, 600 MHz)	23
**Figure S28.** HR-ESI-MS spectrum of **8** showing characteristic isotopic pattern of hydrogen-deuterium exchange	23
**Figure S29.** HR-ESI-MS/MS spectrum of **9**	24
**References**	24

Table S1. Major ions and their putative annotations of compounds detected in the *F. vesiculosus n*-hexane subextract. Annotations were based on GNPS and *in silico* MS/MS databases, as well as manual dereplication of m/z ($[M+H]^+$ or $[M+Na]^+$), retention time (t_R), fragmentation pattern and predicted molecular formula (< 5 ppm) against Dictionary of Marine Natural Products, MarinLit and DEREP_NP and other literature data. Confidence levels of putative identification after Sumner et al. [1] and Blaženovic et al. [2] are given and specific resources used for annotation are provided in superscript (for key see footnote). (I.D.: **1–7** = compound I.D. for isolated compounds**, h1–h23** = compound I.D. for compounds dereplicated from the *n*-hexane subextract)

I.D.	*m/z*	t_R (min)	Present in fraction	MS/MS (*m/z*)	Ion type	Molecular formula	Identified compound	Compound class	Confidence	Reference
	520.3045	1.46	H47	180		$C_{30}H_{43}NO_5$			4	
	461.2892	3.08	H47	-		$C_{27}H_{40}O_6$			4	
**h1**	641.4215	3.59	H32 - 34	581; 411; 275; 149; 109	$[M-H_2O+H]^+$	$C_{42}H_{56}O_5$	Fucoxanthin (dehyd.)	Carotenoid	2*L	[3]
	609.2728		H47	591; 559; 531;		$C_{34}H_{40}O_{10}$			4	
**h2**	593.2769	3.681	H44, H47	533	$[M+H]^+$	$C_{35}H_{36}N_4O_5$	Pheophorbide A	Tetrapyrrole/ Chlorophyll	2I, L, D	[4]
**h1**	681.4148	4.02	H31, H33, H34	663, 603; 589; 527; 509; 449	$[M+Na]^+$	$C_{42}H_{58}O_6$	Fucoxanthin	Carotenoid	2*L, D	[3]
**h3**	981.5572		H47	819; 705; 679	$[M+Na]^+$	$C_{53}H_{82}O_{15}$	DGDG (20:5/18:4)	Galactolipid	2*L	[5]
**7**	983.5672	4.56	H47	821; 705; 681	$[M+Na]^+$	$C_{53}H_{84}O_{15}$	DGDG (20:5/18:3)	Galactolipid	2P	[5]
	607.2930	5.02	H25, H26	547		$C_{26}H_{48}O_{14}$			4	
**2**	793.4849	5.55	H42	517	$[M+Na]^+$	$C_{45}H_{70}O_{10}$	MGDG (18:4/18:4)	Galactolipid	1P	[5]
**1**	819.5018	5.56	H39 - 44	517; 543	$[M+Na]^+$	$C_{47}H_{74}O_{10}$	MGDG (20:5/18:3)	Galactolipid	1P	[3,5]
	549.2868	5.79	H25	521; 461		$C_{24}H_{46}O_{12}$			4	
**3**	795.5009	6.14	H40 - 42	517; 519	$[M+Na]^+$	$C_{45}H_{72}O_{10}$	MGDG (18:3/18:4)	Galactolipid	1P	[5]
**5**	821.5193	6.30	H39 - 42	519; 543	$[M+Na]^+$	$C_{47}H_{74}O_{10}$	MGDG (20:5/18:3)	Galactolipid	1P	[5]
**4**	797.5190	6.34	H41, H42	519	$[M+Na]^+$	$C_{45}H_{74}O_{10}$	MGDG (18:3/18:3)	Galactolipid	1P	[5]
**6**	821.5180	6.65	H40 - 42	517; 545	$[M+Na]^+$	$C_{47}H_{74}O_{10}$	MGDG (20:4/18:4)	Galactolipid	1P	[5]
	745.4877	6.84	H40	657; 517; 377		$C_{41}H_{70}O_{10}$		Galactolipid	3**	
**h4**	823.5413	6.85	H40	519; 545	$[M+Na]^+$	$C_{47}H_{76}O_{10}$	MGDG (20:4/18:3)	Galactolipid	2*L	[5]
**h5**	823.5360	7.11	H41, H42	521; 543	$[M+Na]^+$	$C_{47}H_{76}O_{10}$	MGDG (20:5/18:2)	Galactolipid	2*L	[5]
	823.5254	7.23	H43, H44							
**h6**	799.5363	7.24	H41, H42	519; 521	$[M+Na]^+$	$C_{45}H_{76}O_{10}$	MGDG (18:3/18:2)	Galactolipid	2*L	[5]

	419.3520	7.25	H24-26		$[M+H]^+$	$C_{26}H_{42}O_4$		Lipid	3[I]	
**h7**	849.5517	7.44	H40 - 42	553	$[M+Na]^+$	$C_{49}H_{78}O_{10}$	MGDG (20:4/20:4)	Galactolipid	2*[L]	[5]
	773.5189	7.50	H40	685; 517; 405		$C_{43}H_{74}O_{10}$		Galactolipid	3**	
**h8**	825.5535	7.59	H41, H42	521; 545	$[M+Na]^+$	$C_{47}H_{78}O_{10}$	MGDG (20:4/18:2)	Galactolipid	2*[L]	[5]
	439.3190	7.66	H13, H15	173		$C_{27}H_{44}O_3$			4	
	689.4974	7.77	H24	377		$C_{39}H_{70}O_8$			4	
**h9**	825.5532	7.81	H39, H42	523; 543	$[M+Na]^+$	$C_{47}H_{78}O_{10}$	MGDG (20:5/18:1)	Galactolipid	2*[L]	[5]
**h10**	773.5192	7.83	H37, H38	685; 649; 517; 377	$[M+Na]^+$	$C_{43}H_{74}O_{10}$	MGDG (18:4/16:0)	Galactolipid	2*[L]	[5]
	627.4623	7.87	H21 - 31	371		$C_{37}H_{64}O_6$			4	
	453.3346	7.89	H13, H15, H16	187		$C_{28}H_{46}O_3$			4	
**h11**	801.5505	7.96	H40 - 42	519; 523	$[M+Na]^+$	$C_{45}H_{78}O_{10}$	MGDG (18:3/18:1)	Galactolipid	2*[L]	[5]
**h12**	769.5047	8.01	H34	465	$[M+Na]^+$	$C_{43}H_{70}O_{10}$	MGDG (20:4/14:2)	Galactolipid	2*[L]	[5]
	693.4711	8.14	H21 - 33, H35, H37 - 40	465; 391; 303		$C_{41}H_{66}O_7$			4	
**h13**	827.5672	8.28	H40 - 42	523; 545	$[M+Na]^+$	$C_{47}H_{80}O_{10}$	MGDG (20:4/18:1)	Galactolipid	2*[L]	[5]
	745.5017	8.39	H37, H38	441		$C_{47}H_{68}O_7$		Galactolipid	3**	
**h14**	751.5365	8.57	H41 - 43	523; 469	$[M+Na]^+$	$C_{41}H_{76}O_{10}$	MGDG (18:1/14:0)	Galactolipid	2*[L]	[5]
	669.4719	8.59	H21 - 33, H35, H37 - 40	441; 391		$C_{39}H_{66}O_7$			4	
**h15**	777.5507	8.72	H41 - 43	521; 497	$[M+Na]^+$	$C_{43}H_{78}O_{10}$	MGDG (18:2/16:0)	Galactolipid	2*[L]	[5]
	695.4866	8.78	H37, H38	391; 467		$C_{41}H_{69}O_7$			4	
**h16**	429.3742	8.88	H10 - 13	165	$[M+H]^+$	$C_{29}H_{48}O_2$	α-Tocomonoenol	Vitamin/ Terpenoid	2[I]	
**h17**	431.3812	8.98	H10	165	$[M+H]^+$	$C_{29}H_{50}O_2$	α-Tocopherol	Vitamin/ Terpenoid	2[G]	[6]
	695.4872	9.08	H21 - 33, H35, H37 - 42	391; 467		$C_{41}H_{69}O_7$			4	
	893.5584		H18	615; 539					4	
**h18**	871.5760	9.10	H18, H19	593; 592; 533	$[M+H]^+$	$C_{55}H_{74}N_4O_5$	Pheophytin A	Chlorophyll	2*[L, D]	[4]
	671.4882		H21 - 34, H36 - 41	391; 443		$C_{39}H_{68}O_7$			4	

**h19**	697.5033	9.19	H37, H38	469; 391	$[M+Na]^+$	$C_{33}H_{54}O_{14}$	DGMG (18:4)	Galactolipid	2*L	[5]
**h20**	779.5656	9.38	H39 - 43	523; 497	$[M+Na]^+$	$C_{43}H_{80}O_{10}$	MGDG (18:1/16:0)	Galactolipid	2*L	[5]
**h21**	697.5054	9.44	H21 - 31, H34	469; 441; 419	$[M+Na]^+$	$C_{33}H_{54}O_{14}$	DGMG (18:4)	Galactolipid	2*L	[5]
	723.5188	9.75	H22 - 28	467; 419		$C_{43}H_{72}O_7$		Galactolipid	3**	
	673.5028	9.98	H19, H21 - 34, H37 - 40	391; 445		$C_{39}H_{70}O_7$			4	
**h22**	699.5188	10.07	H21 - 32, H34, H39, H40	443; 419	$[M+Na]^+$	$C_{33}H_{56}O_{14}$	DGMG (18:3)	Galactolipid	2*L	[5]
**h23**	885.5909	12.92	H18, H19	607, 580, 533	$[M+H]^+$	$C_{55}H_{72}N_4O_6$	Pheophytin B	Chlorophyll	2*,L	

* Experimental MS/MS fragment data matched with known compound data

** Compound class annotated based on similar fragment ions observed in related ions

G Annotation based on GNPS MS/MS database match (this type of match shows a confirmed MS/MS fragment match with a known compound, thus it is not also labelled *)

I Annotation based on *in silico* UNPD MS/MS database match (this type of match shows a confirmed MS/MS fragment match with a known compound, thus it is not also labelled *)

D Molecular formula of annotated compound matched in database

P Compound was isolated in pure state and structure elucidated based on NMR and MS data

L Annotation matched to relevant literature data

Table S2. Major ions and their putative annotations of compounds detected in the *F. vesiculosus n*-BuOH subextract. Annotations were based on GNPS and *in silico* MS/MS databases, as well as manual dereplication of *m/z* ([M+H]-), retention time (t_R), fragmentation pattern and predicted molecular formula (< 5 ppm) against Dictionary of Marine Natural Products, MarinLit and DEREP_NP and other literature data. Confidence level of putative identification after Sumner et al. [1] and Blaženovic et al. [2]. (I.D.: **8–9** = compound I.D. for isolated compounds**, b1–b22** = compound I.D. for compounds dereplicated from the *n*-BuOH subextract)

I.D.	m/z	t_R (min)	Present in fraction	MS/MS (m/z)	Ion type	Molecular formula	Identified compound	Compound class	Confidence	Reference
**b1**	181.0700	0.45	B2 - B7	163; 136; 119; 101; 89	[M-H]-	$C_6H_{14}O_6$	Galacitol / mannitol	Sugar	2G	[7]
**b2**	223.0806	0.753	B5 - B8	181; 163; 126; 101	[M-H]-	$C_8H_{16}O_7$	*D*-6-*O*-Acetylglucose	Sugar	2 G	
**b3**	497.0717	0.90	B11, B12	461; 435; 393; 367; 349; 331; 287; 245; 165	[M-H]-	$C_{24}H_{18}O_{12}$	Tetrafucol	Phlorotannin (tetramer)	3*L	[8]
	497.2475	1.09	B9 - B12	-	[M-H]-	$C_{24}H_{18}O_{12}$		Phlorotannin**	3**	
**b3**	497.0721	1.09	B5 - B14	461; 435; 393; 367; 349; 331; 287; 245; 165	[M-H]-	$C_{24}H_{18}O_{12}$	Tetrafucol	Phlorotannin	2*L	[9]
**b4**	621.0890	1.42	B12, B13	585; 559; 517; 478; 461; 411; 251; 331; 289; 254; 207; 165; 139; 125; 97	[M-H]-	$C_{30}H_{22}O_{15}$	Trifucophlorethol	Phlorotannin	2*L	[10]
**b5**	373.0560	1.45	B13	355; 329; 311; 231; 189; 141; 139	[M-H]-	$C_{18}H_{14}O_9$	Trifucol	Phlorotannin	3*L	[9]
**b6**	745.1033	1.64	B13	709; 602; 584; 558; 433; 331; 287; 269; 165; 125	[M-H]-	$C_{36}H_{26}O_{18}$	Hexafucol	Phlorotannin	2*L	[9]
**8**	957.1215	1.82	B15	921; 877; 773; 477; 461; 435; 431; 391; 353; 313	[M-H]-	$C_{48}H_{30}O_{22}$		Phlorotannin**	2/3P	
**b7**	373.0564	1.96	B13 - B15	247; 229; 201; 189; 161; 139; 125	[M-H]-	$C_{18}H_{14}O_9$	Triphlorethol	Phlorotannin (phlorethol)	3*L	[11]
**b8**	479.0607	2.142	B11 - B16	461; 435; 417; 393; 375; 367; 349; 337; 313; 285	[M-H]-	$C_{24}H_{16}O_{11}$	Fucofurodiphlorethol	Phlorotannin	3*L	[8]
	1007.1541	2.14	B11, B12	509; 491; 473; 461; 435; 393; 353; 331; 309; 287; 254; 205; 179; 177; 165; 137	[M-H]-	$C_{56}H_{32}O_{19}$, $C_{54}H_{32}O_{19}$, $C_{58}H_{32}O_{16}$, $C_{60}H_{32}O_{16}$		Phlorotannin**	3**	
**9**	497.0733	2.31	B11 - B15	327; 265; 247; 233; 229; 215; 203; 139; 123	[M-H]-	$C_{24}H_{18}O_{12}$	Fucodiphloroethol	Phlorotannin (phlorethol)**	2*L	[8]

	603.0794	2.35	B15	585; 559; 541; 517; 491	$[M-H]^-$			Phlorotannin**	3**	
	869.119	2.581	B14, B15	Very low MS/MS	$[M-H]^-$	$C_{42}H_{30}O_{21}$		Phlorotannin**	3**	
	635.1040	2.37	B12	497; 479; 461; 435; 393; 353; 331; 205; 165; 125; 109	$[M-H]^-$	$C_{31}H_{24}O_{15}$, $C_{29}H_{24}O_{15}$, $C_{22}H_{28}O_{20}$, $C_{24}H_{28}O_{20}$		Phlorotannin**	3**	
**b9**	993.1359	2.58	B15, B16	Very low MS/MS	$[M-H]^-$	$C_{48}H_{34}O_{25}$	Pentafucodiphlorethol	Phlorotannin**	3*[L]	[8]
	571.0731	2.69	B12	449; 393; 364; 349; 339; 305; 165	$[M-H]^-$	$C_{26}H_{20}O_{15}$		Phlorotannin**	3**	
	745.1072	2.81	B14 - B17	479; 365; 355; 309; 247; 245; 229; 139; 123	$[M-H]^-$	$C_{36}H_{26}O_{18}$		Phlorotannin	3**	
**b10**	719.3498	2.81	B25-29	415; 397; 275;171; 143; 119; 89	$[M-H]^-$	$C_{38}H_{73}O_{10}P$	PG (32:1)	Phospholipid	2*[L]	[5]
**b11**	787.3362	2.88	B26-27	112	$[M-H]^-$	$C_{41}H_{72}O_{12}S$	SQDG (18:3/14:0)	Sulfolipid	2*[L]	[5]
	671.3498	6.84	B25, B26	415; 397; 305; 277; 119	$[M-H]^-$			Phospholipid**	3**	
**b12**	721.3654	7.242	B26-29	415; 397; 277; 171; 119	$[M-H]^-$	$C_{38}H_{75}O_{10}P$	PG (32:0)	Phospholipid	2*[L]	[5]
**b13**	789.3599	6.96	B27	397; 277; 112	$[M-H]^-$	$C_{41}H_{74}O_{12}S$	SQDG (18:2/14:0)	Sulfolipid	2*[L]	[5]
	527.4341	7.08	B29	-	$[M-H]^-$	$C_{23}H_{43}O_{11}S$			4	
**b14**	527.2532	7.24	B22 - 29	225	$[M-H]^-$	$C_{23}H_{43}O_{11}S$	SQMG (14:0)	Sulfolipid	2*[L]	[5]
**b15**	577.2690	7.36	B24 - B29	299; 225;164; 134; 80	$[M-H]^-$	$C_{75}H_{45}O_{11}S$	SQMG (18:3)	Sulfolipid	2*[L]	[5]
	699.3813	7.49	B26-29	415; 397; 255; 119	$[M-H]^-$			Phospholipid**	3**	
**b16**	500. 2776	7.50	B26-29	303; 259; 196	$[M-H]^-$	$C_{25}H_{44}NO_7P$	LPE (20:4)	Phospholipid	2[G]	[5]
	725.3973	7.69	B26-29	415; 397; 281; 119; 89	$[M-H]^-$			Phospholipid**	3**	
**b17**	555.4699	8.07	B27, B29	299; 255; 225; 164; 134	$[M-H]^-$	$C_{25}H_{47}O_{11}S$	SQMG (16:0)	Sulfolipid	2*[L]	[5]
**b17**	555.2843	8.07	B20 - B27	299; 255; 225; 206; 164	$[M-H]^-$	$C_{25}H_{47}O_{11}S$	SQMG (16:0)	Sulfolipid	2*[L]	[5]
**b18**	597.3046	8.33	B28-29	315; 281; 241; 153	$[M-H]^-$	$C_{27}H_{51}O_{12}P$	LPI (18:1)	Lyso-phospholipid	2*[L]	[5]

* Experimental MS/MS fragment data matched with known compound data ** Compound class annotated based on similar fragment ions observed in related ions

[G] Annotation based on GNPS MS/MS database match (this type of match shows a confirmed MS/MS fragment match with a known compound, thus it is not also labelled *)

[I] Annotation based on *in silico* UNPD MS/MS database match (this type of match shows a confirmed MS/MS fragment match with a known compound, thus it is not also labelled *)

[D] Molecular formula of annotated compound matched in database [P] Compound was isolated in pure state and structure elucidated based on NMR and MS data

[L] Annotation matched to relevant literature data

Table S3. ^{1}H NMR (400 MHz) data of galactolipids **1 – 6** in $CDCl_3$ (δ in ppm)

Position	**1**	**2**	**3**	**4**	**5**	**6**
	δ_H, Mult. (*J* in Hz)	δ_H, Mult. (*J* in Hz)	δ_H, Mult. (*J* in Hz)	δ_H, Mult. (*J* in Hz)	δ_H, Mult. (*J* in Hz)	δ_H, Mult. (*J* in Hz)
**1**	4.40 dd (12.1, 3.5)	4.39 dd (12.1, 3.5)	4.39 dd (12.1, 3.5)	4.39 dd (12.1, 3.5)	4.40 dd (12.1, 3.5)	4.39 dd (12.1, 3.5)
	4.21 dd (12.1, 6.5)	4.21 dd (12.1, 6.5)	4.21 dd (12.1, 6.5)	4.21 dd (12.1, 6.5)	4.21 dd (12.1, 6.5)	4.21 dd (12.1, 6.5)
**2**	5.31 m	5.28 m	5.28 m	5.28 m	5.30 m	5.28 m
**3**	3.90 dd (12.1, 5.5)	3.90 dd (12.1, 5.5)	3.90 dd (11.5, 5.5)	3.90 dd (11.5, 5.5)	3.90 dd (11.5, 5.5)	3.90 dd (11.5, 5.5)
	3.75 dd (11.3, 6.3)	3.75 dd (11.6, 6.0)	3.75 dd (11.5, 6.3)	3.75 dd (11.5, 6.3)	3.75 dd (11.5, 6.3)	3.75 dd (11.5, 6.3)
**2'**	2.33 m	2.35 m	2.33 m	2.33 m	2.33 m	2.33 m
**3'**	1.69 m	1.65 m	1.69 m	1.50 - 1.69	1.69 m	1.69 m
**4'**	2.07 m	1.38 m	1.29–1.32	1.27–1.35	2.07 m	1.29–1.32
**5'**	5.31–5.39	2.07 m	1.29–1.32	1.27–1.35	5.31–5.39	1.29–1.32
**6'**	5.31–5.39	5.31–5.39	1.29–1.32	1.27–1.35	5.31–5.39	1.29–1.32
**7'**	2.81 m	5.31–5.39	1.29–1.32	1.27–1.35	2.81 m	2.07 m
**8'**	5.31–5.39	2.81 m	2.07 m	2.07 m	5.31–5.39	5.31–5.39
**9'**	5.31–5.39	5.31–5.39	5.31–5.39	5.31–5.39	5.31–5.39	5.31–5.39
**10'**	2.81 m	5.31–5.39	5.31–5.39	5.31–5.39	2.81 m	2.81 m
**11'**	5.31–5.39	2.81 m	2.81 m	2.80 m	5.31–5.39	5.31–5.39
**12'**	5.31–5.39	5.31–5.39	5.31–5.39	5.31–5.39	5.31–5.39	5.31–5.39
**13'**	2.81 m	5.31–5.39	5.31–5.39	5.31–5.39	2.81 m	2.81 m
**14'**	5.31–5.39	2.81 m	2.81 m	2.80 m	5.31–5.39	5.31–5.39
**15'**	5.31–5.39	5.31–5.39	5.31–5.39	5.31–5.39	5.31–5.39	5.31–5.39
**16'**	2.81 m	5.31–5.39	5.31–5.39	5.31–5.39	2.80 m	2.81 m
**17'**	5.31–5.39	2.08 m	2.08 m	2.07 m	5.31–5.39	5.31–5.39
**18'**	5.31–5.39	0.97 t (7.5)	0.97 t (7.5)	0.97 t (7.5)	5.31–5.39	5.31–5.39
**19'**	2.08 m				2.07 m	2.08 m
**20'**	0.97 t (7.5)				0.97 t (7.5)	0.97 t (7.5)
**2''**	2.35 m	2.35 m	2.35 m	2.35 m	2.33 m	2.35 m
**3''**	1.65 m	1.65 m	1.65 m	1.50 - 1.69	1.50 - 1.69	1.65 m
**4''**	1.38 m	1.38 m	1.38 m	1.27–1.35	1.27–1.35	1.38 m
**5''**	2.07 m	2.07 m	2.07 m	1.27–1.35	1.27–1.35	2.07 m
**6''**	5.31–5.39	5.31–5.39	5.31–5.39	1.27–1.35	1.27–1.35	5.31–5.39
**7''**	5.31–5.39	5.31–5.39	5.31–5.39	1.27–1.35	1.27–1.35	5.31–5.39
**8''**	2.81 m	2.81 m	2.81 m	2.07 m	2.07 m	2.81 m
**9''**	5.31–5.39	5.31–5.39	5.31–5.39	5.31–5.39	5.31–5.39	5.31–5.39
**10''**	5.31–5.39	5.31–5.39	5.31–5.39	5.31–5.39	5.31–5.39	5.31–5.39
**11''**	2.81 m	2.81 m	2.81 m	2.80 m	2.81 m	2.81 m
**12''**	5.31–5.39	5.31–5.39	5.31–5.39	5.31–5.39	5.31–5.39	5.31–5.39
**13''**	5.31–5.39	5.31–5.39	5.31–5.39	5.31–5.39	5.31–5.39	5.31–5.39
**14''**	2.81 m	2.81 m	2.81 m	2.80 m	2.81 m	2.81 m
**15''**	5.31–5.39	5.31–5.39	5.31–5.39	5.31–5.39	5.31–5.39	5.31–5.39
**16''**	5.31–5.39	5.31–5.39	5.31–5.39	5.31–5.39	5.31–5.39	5.31–5.39
**17''**	2.07 m	2.07 m	2.07 m	2.07 m	2.07 m	2.07 m
**18''**	0.97 t (7.5)	0.97 t (7.5)	0.97 t (7.5)	0.97 t (7.5)	0.97 t (7.5)	0.97 t (7.5)
**1'''**	4.28 d (7.2)	4.28 d (7.2)	4.28 d (7.2)	4.28 d (7.2)	4.28 d (7.2)	4.28 d (7.2)
**2'''**	3.63 dd (9.3, 7.2)	3.63 dd (9.3, 7.2)	3.64 m	3.64 m	3.64 m	3.64 m
**3'''**	3.59 dd (9.3, 3.5)	3.59 dd (9.3, 3.5)	3.59 m	3.60 m	3.60 m	3.60 m
**4'''**	4.01 dd (3.5, 0.6)	4.01 dd [a]	4.02 dd [a]	4.02 dd [a]	4.02 dd [a]	4.02 dd (3.0, 0.8)
**5'''**	3.55 m	3.56 m	3.56 m	3.55 m	3.55 m	3.55 m
**6'''**	3.98 dd (12.1, 6.1)	3.99 dd (12.1, 6.1)	3.99 dd (12.1, 6.1)	3.99 dd (12.1, 6.2)	3.99 dd (12.1, 6.1)	3.99 dd (12.1, 6.1)
	3.86 dd (12.1, 6.1)	3.87 m	3.87 m	3.88 m	3.87 m	3.88 m

[a] coupling constants not recorded due to signal overlap.

Figure S1. MRSA inhibitory activity of A) *F. vesiculosus* crude and subextracts B) *n*-hexane subfractions C) *n*-BuOH subfractions. Positive control (PC) = chloramphenicol, 10 µM

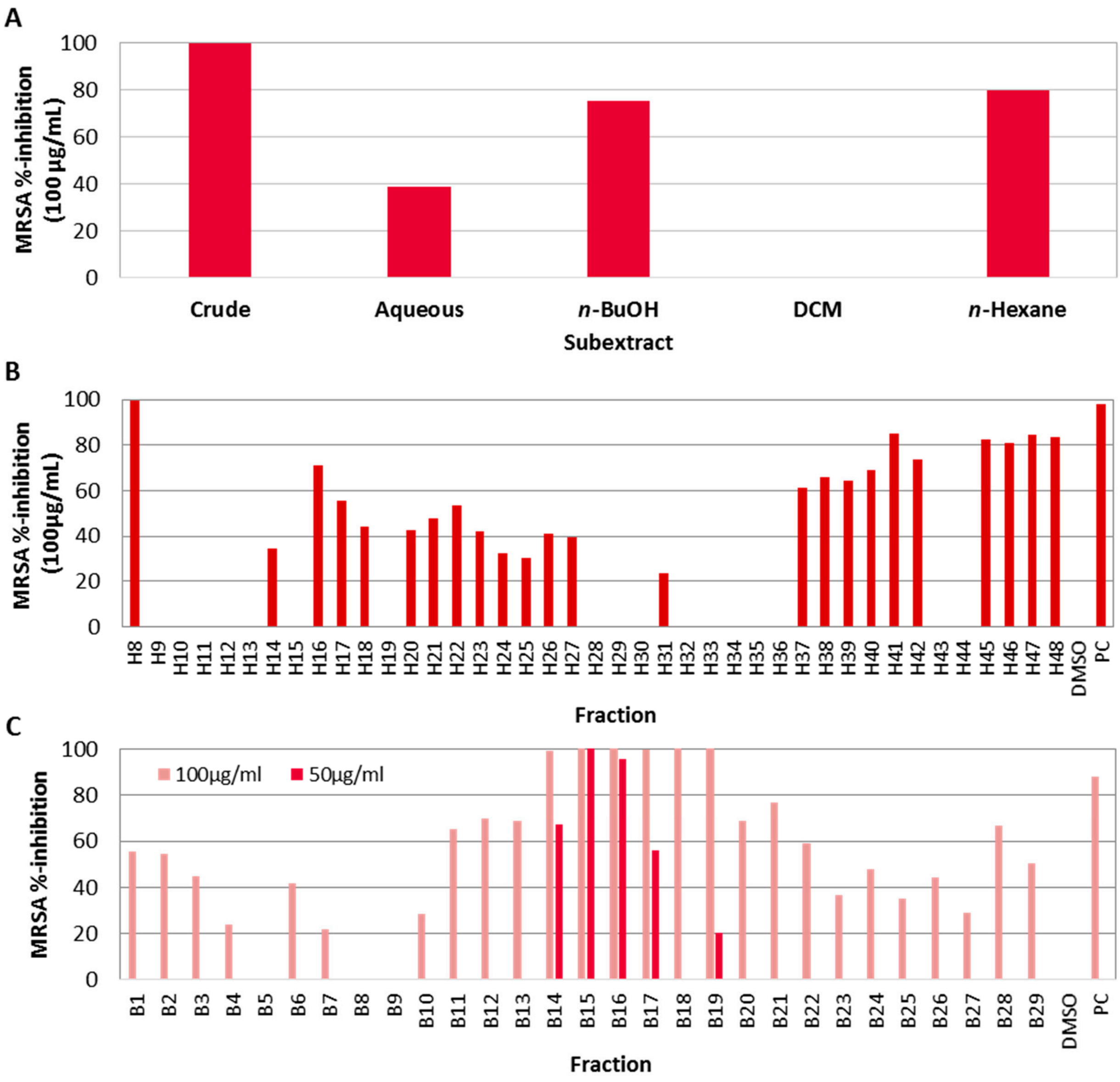

Figure S2. GNPS MS/MS mirror plot of experimental and library data of α-tocopherol (annotation **h17**)

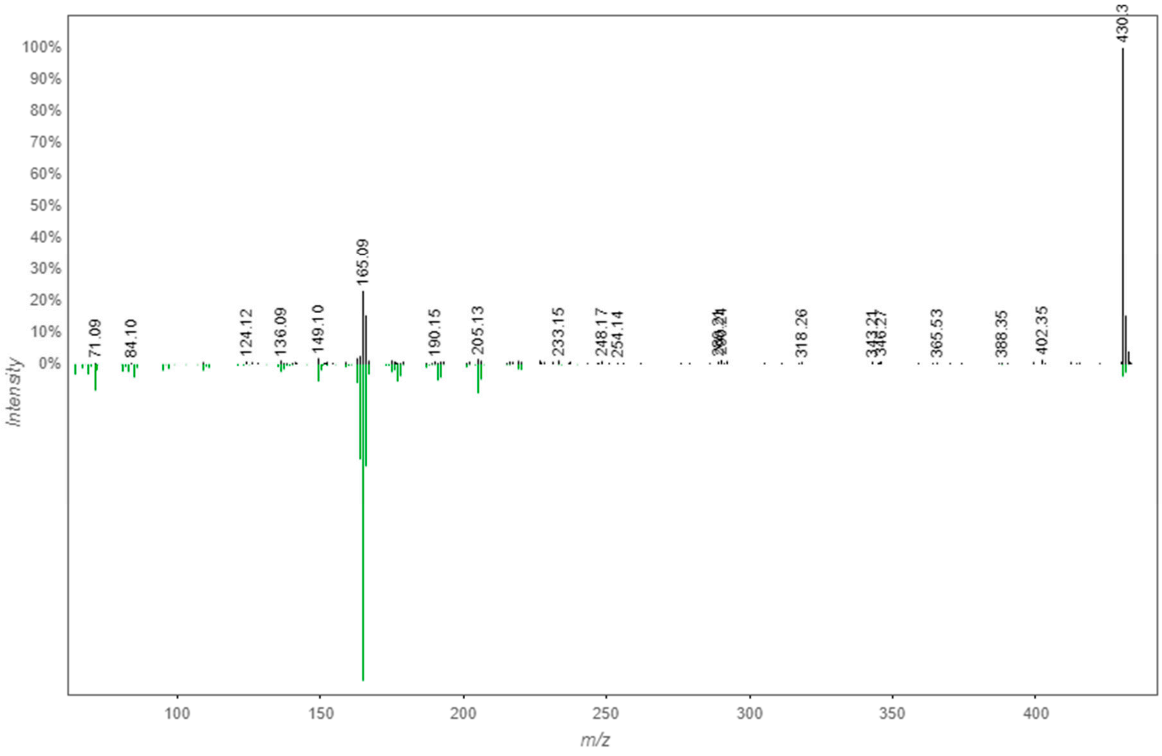

Figure S3. Chemical structures of annotated metabolites in the *F. vesiculosus n*-hexane extract. Numbers correspond to putatively annotated known compounds reported in Table S1

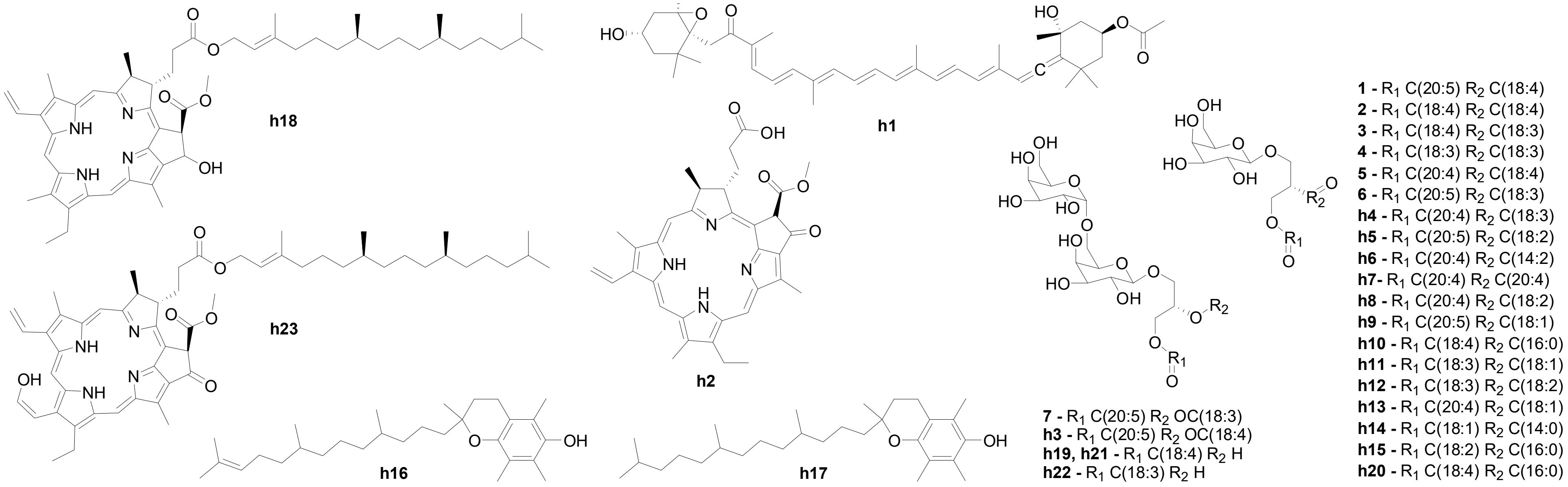

Figure S4. Structure and labelled MS/MS product ions observed in the ESI-MS/MS spectrum of **1** at *m/z* 819.5018 $[M+Na]^+$

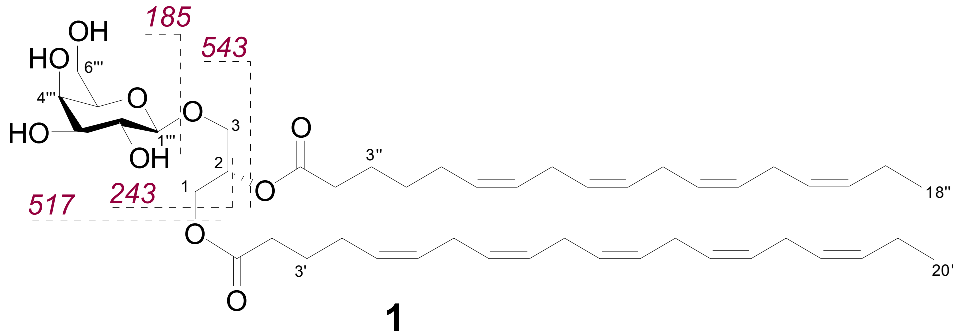

1

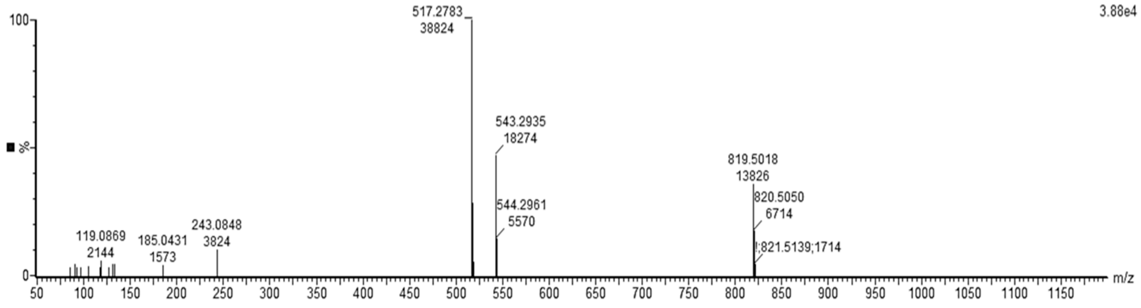

Figure S5. ^{1}H NMR spectrum of **1** ($CDCl_3$, 600 MHz)

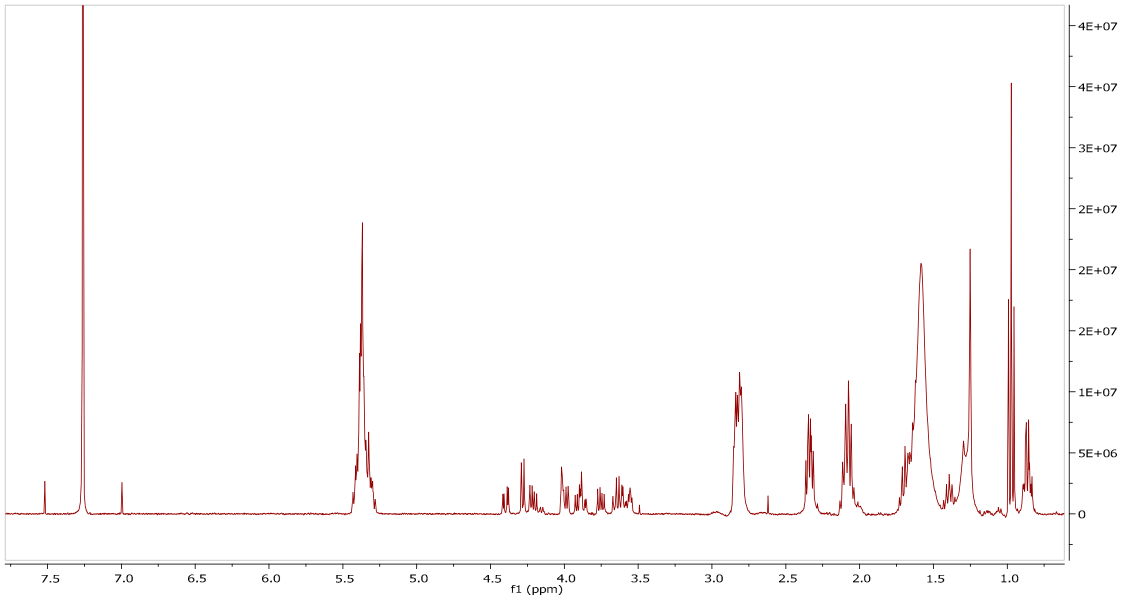

Figure S6. HMBC spectrum of MGDG (20:5/18:4) (**1**) ($CDCl_3$, 600 MHz)

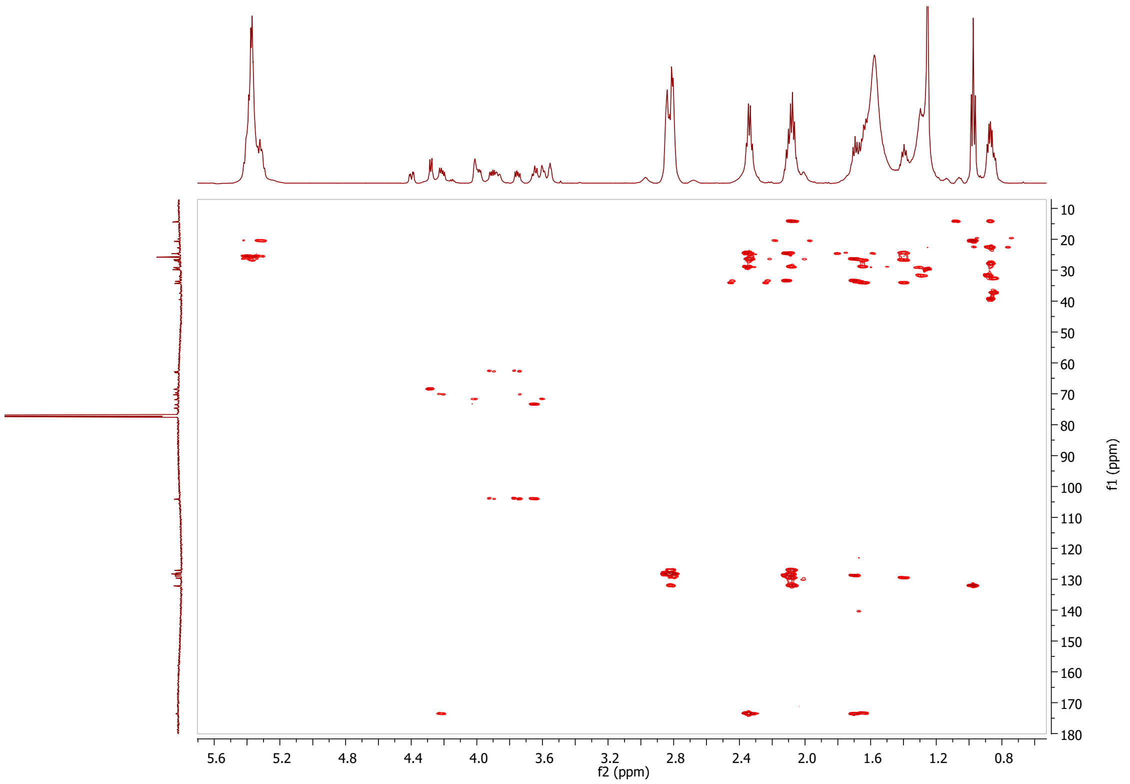

Figure S7. NOESY NMR spectrum of MGDG(20:5/18:4) (**1**) ($CDCl_3$, 600 MHz)

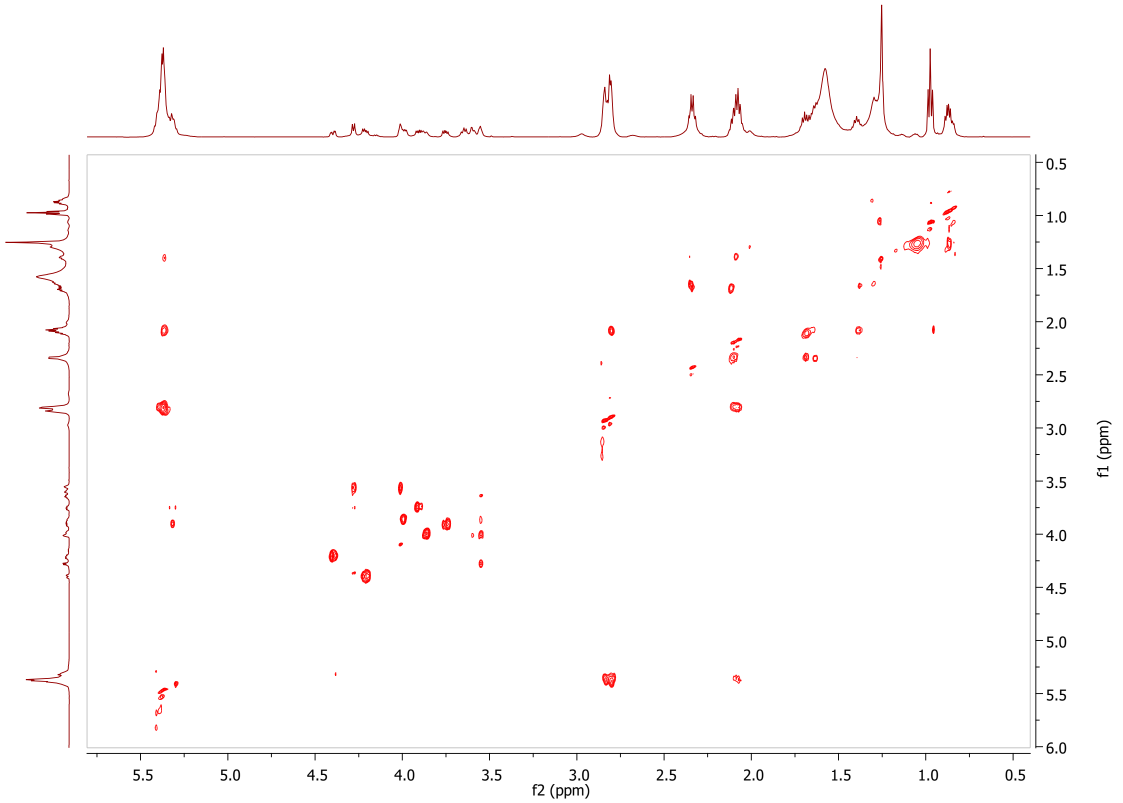

Figure S8. Structure and labelled MS/MS product ions observed in the ESI-MS/MS spectrum of **2** at *m/z* 793.4849 $[M+Na]^+$

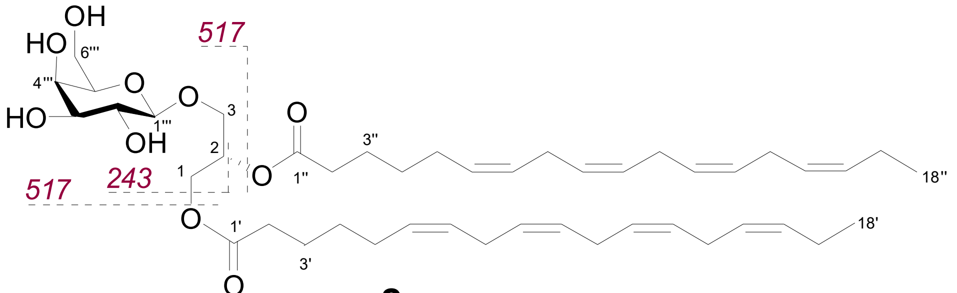

2

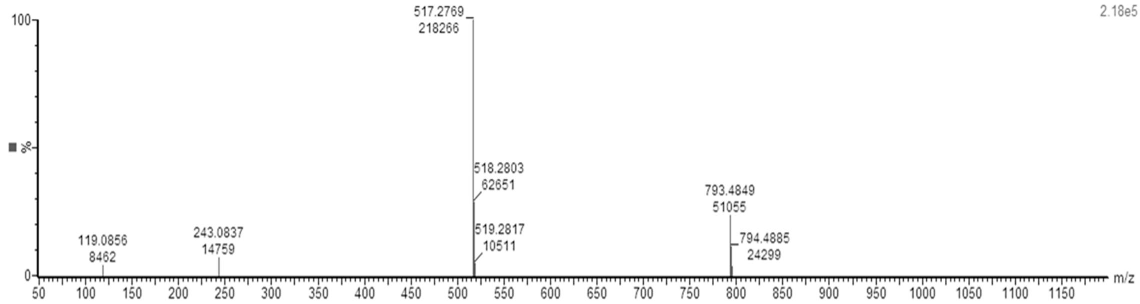

Figure S9. ^1H NMR spectrum of **2** ($CDCl_3$, 400 MHz)

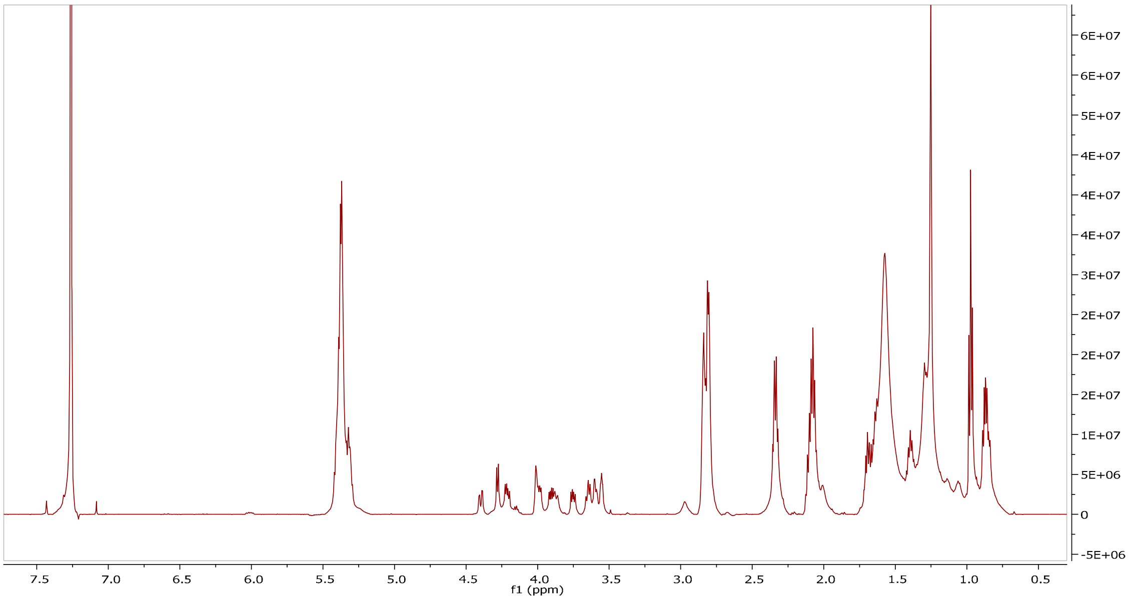

Figure S10. Structure and labelled MS/MS product ions observed in the ESI-MS/MS spectrum of **3** at *m/z* 795.5009 $[M+Na]^+$ ^1H NMR spectrum of **3** ($CDCl_3$, 400 MHz)

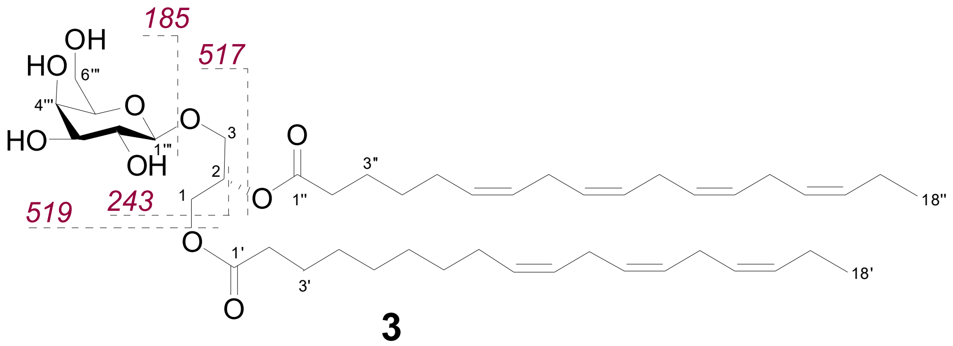

3

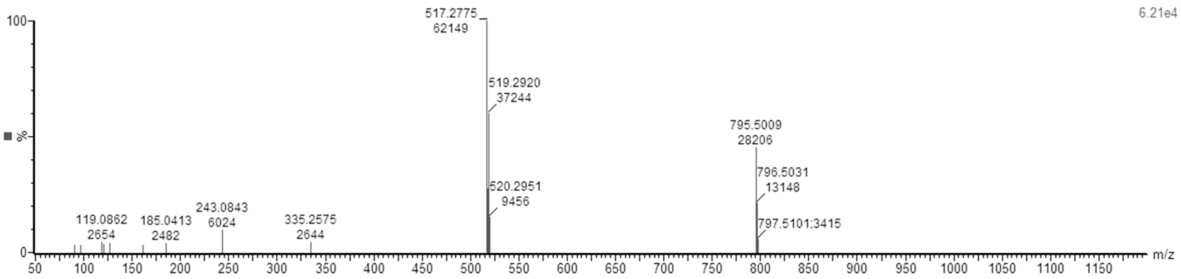

Figure S11. ^1H NMR spectrum of **3** ($CDCl_3$, 400 MHz)

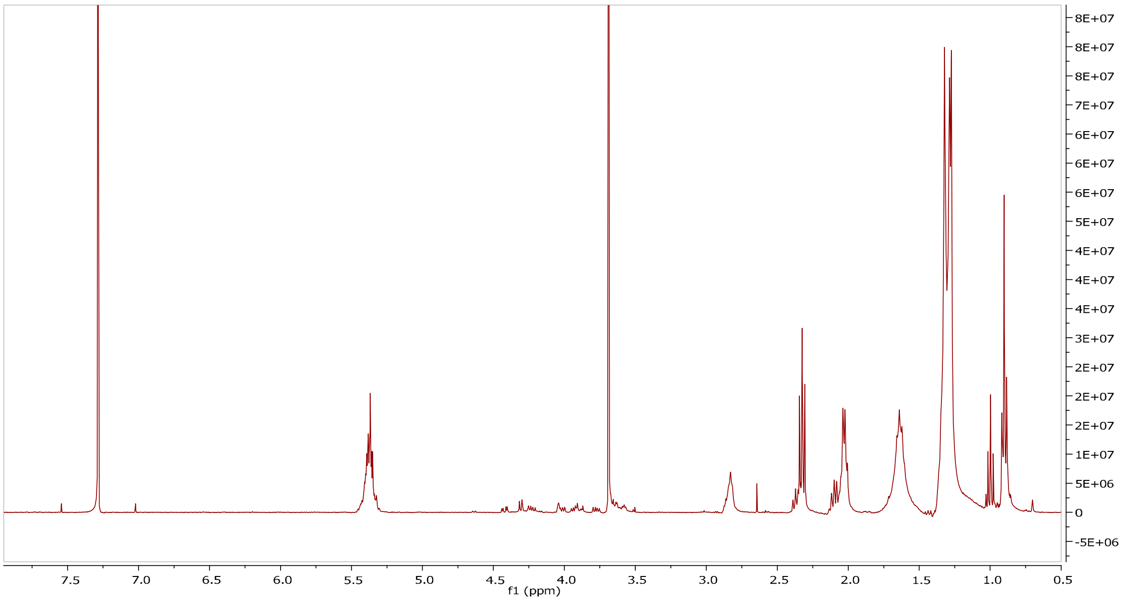

Figure S12. Structure and labelled MS/MS product ions observed in the ESI-MS/MS spectrum of **4** at *m/z* 797.5190 $[M+Na]^+$

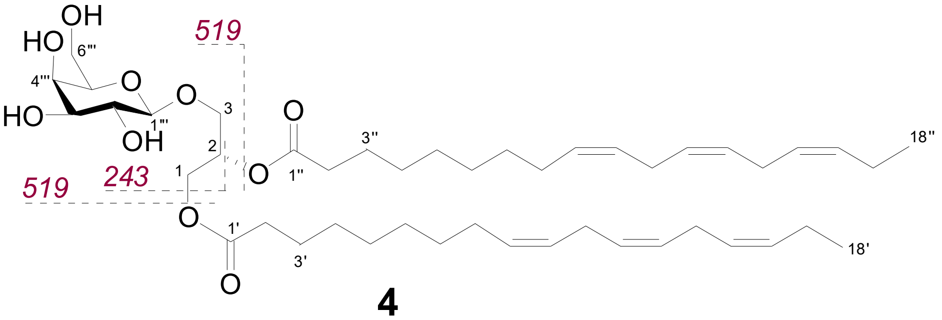

4

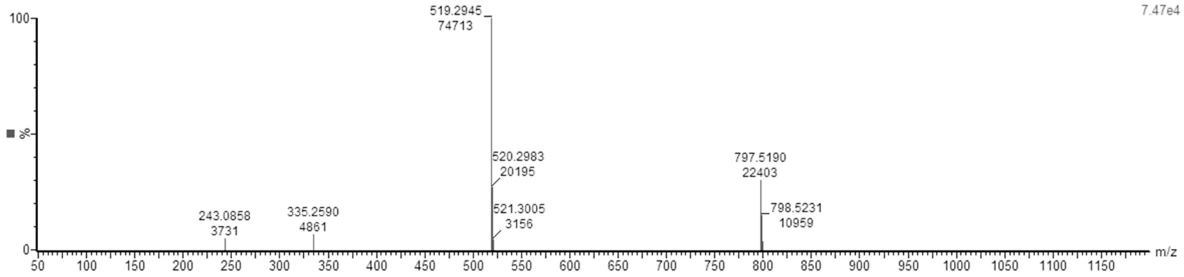

Figure S13. ^{1}H NMR spectrum of **4** ($CDCl_3$, 400 MHz)

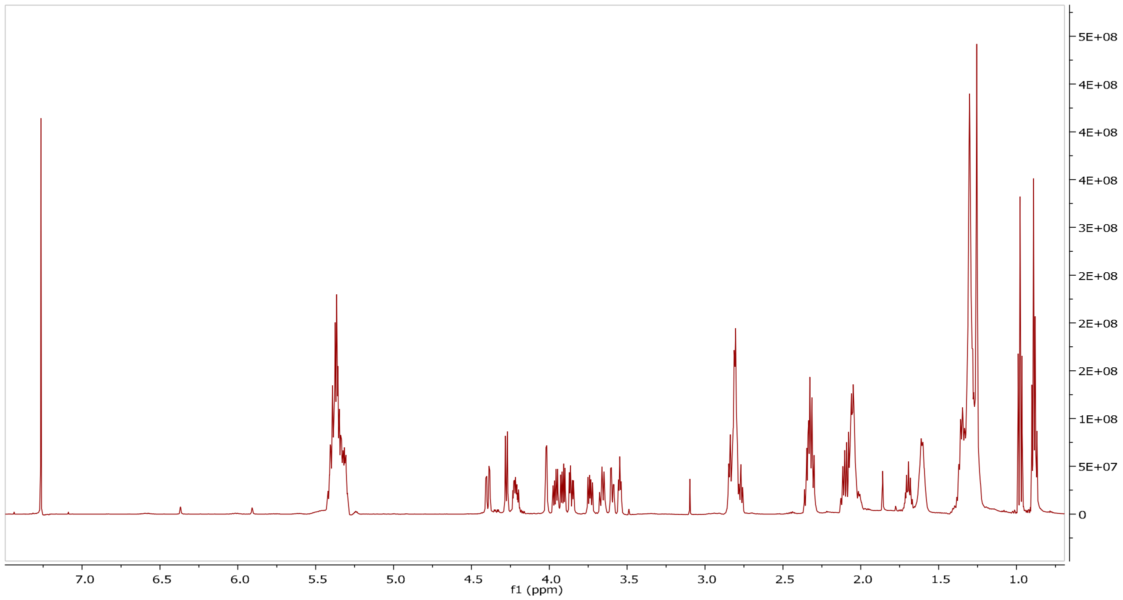

Figure S14. Structure and labelled MS/MS product ions observed in the ESI-MS/MS spectrum of **5** at m/z 821.5180 $[M+Na]^+$

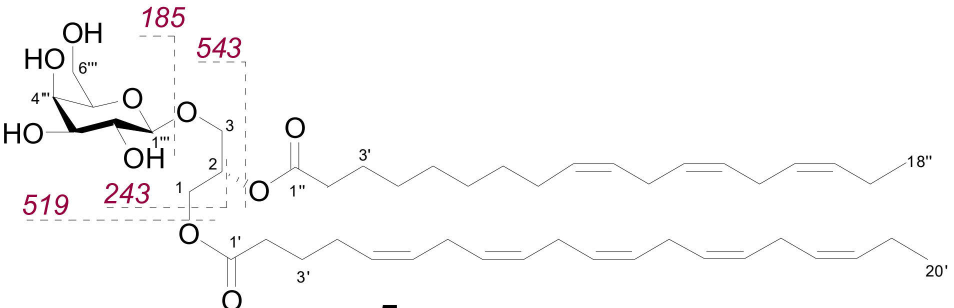

5

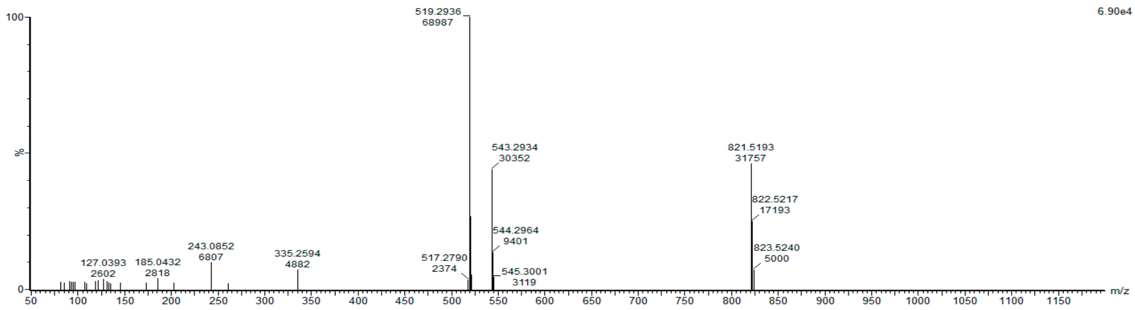

Figure S15. ^1H NMR spectrum of **5** ($CDCl_3$, 400 MHz)

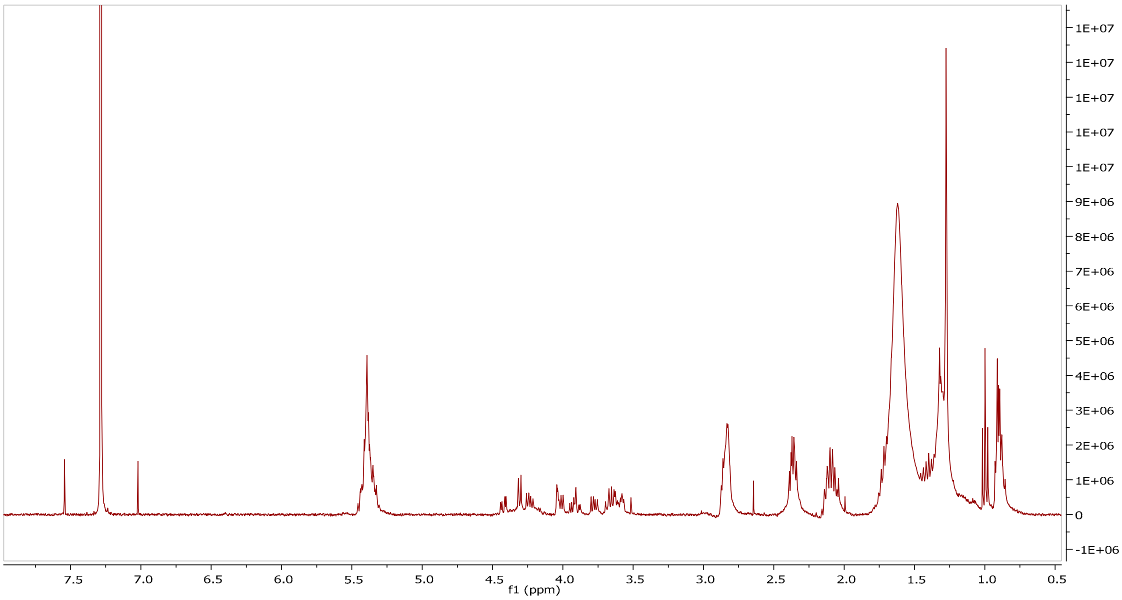

Figure S16. Structure and labelled MS/MS product ions observed in the ESI-MS/MS spectrum of **6** at *m*/*z* 821.5193 [M+Na]+

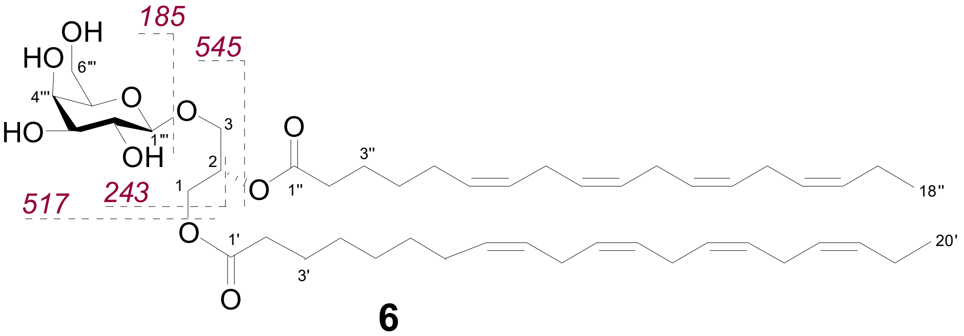

6

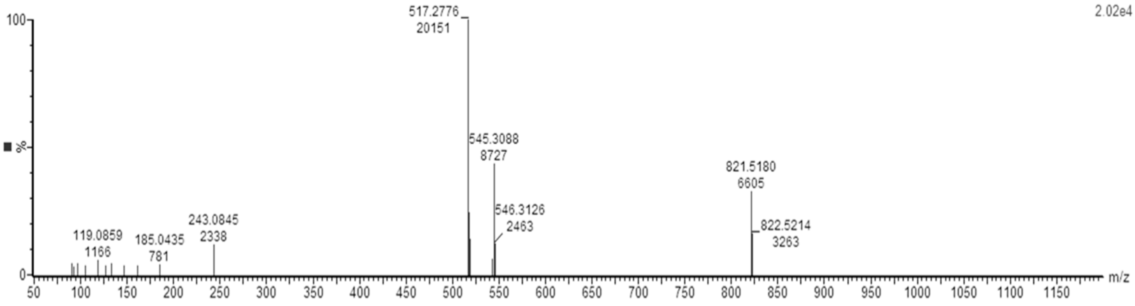

Figure S17. ^{1}H NMR spectrum of **6** ($CDCl_3$, 400 MHz)

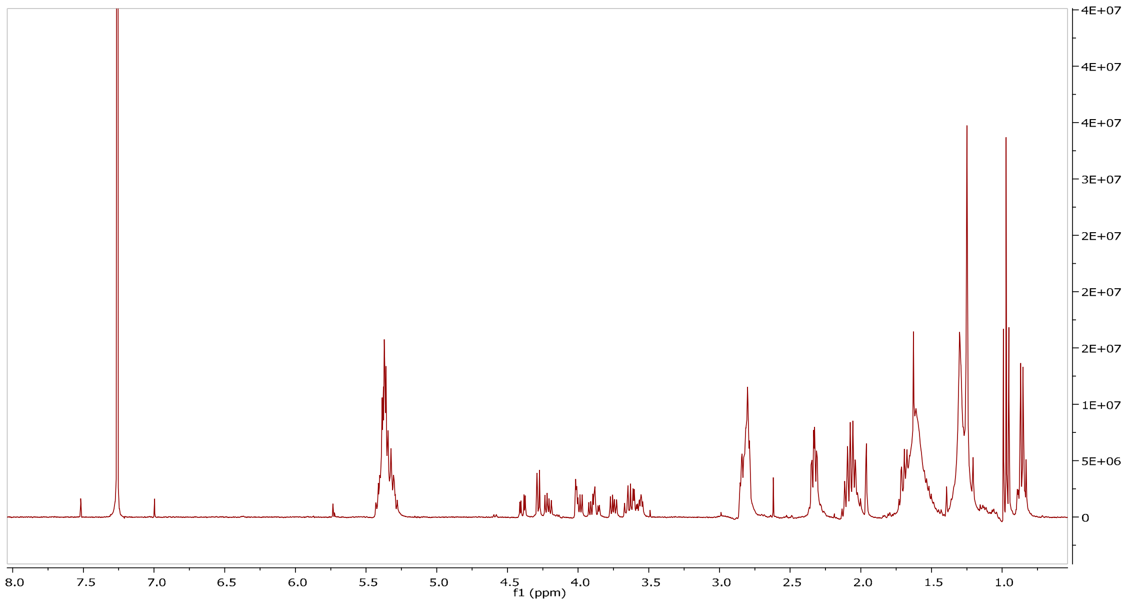

Figure S18. ^1H NMR spectrum of **7** ($CDCl_3$, 400 MHz)

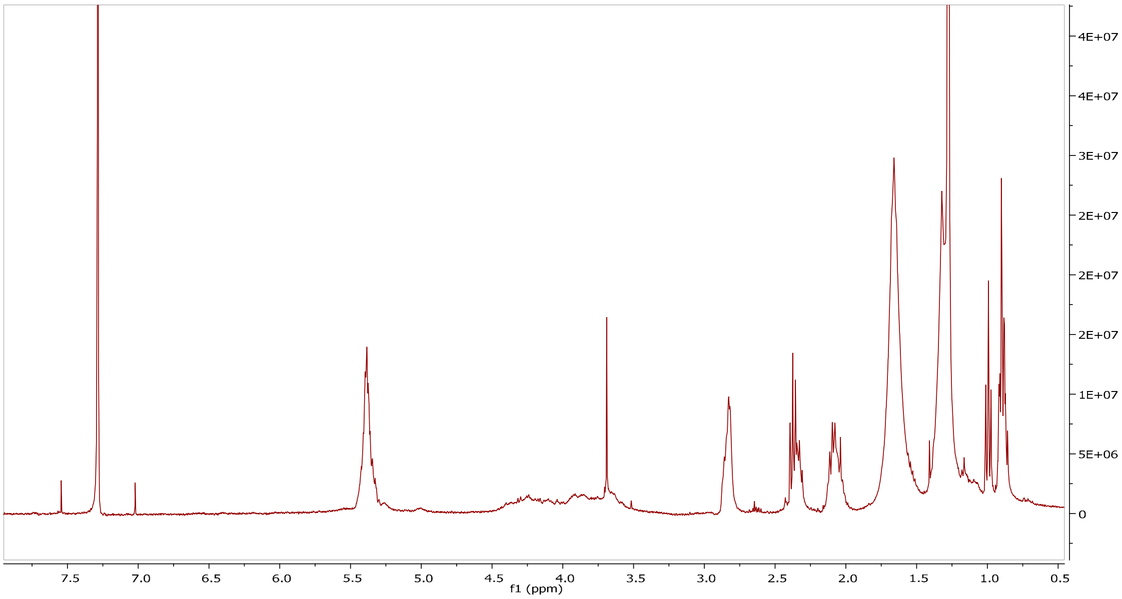

Figure S19. Structure and labelled MS/MS product ions observed in the ESI-MS/MS spectrum of **7** at *m/z* 983.5672 [M+Na]$^+$

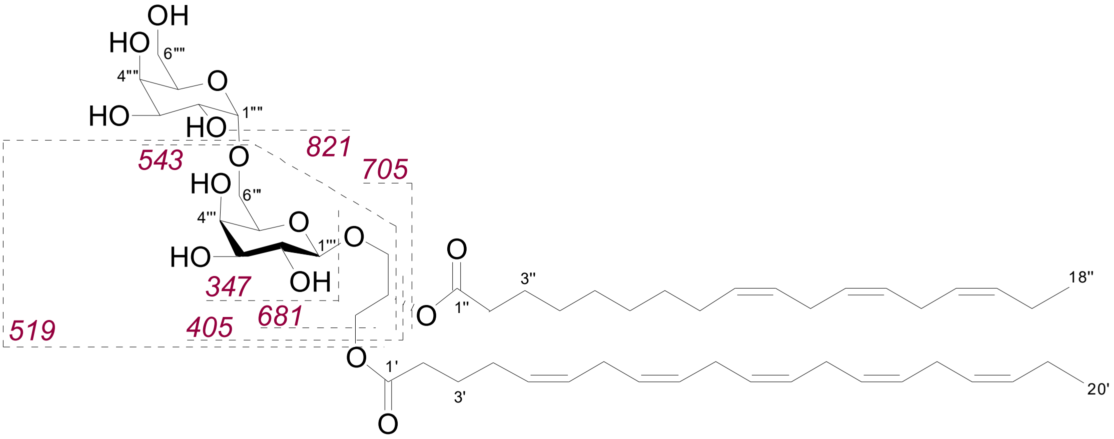

7

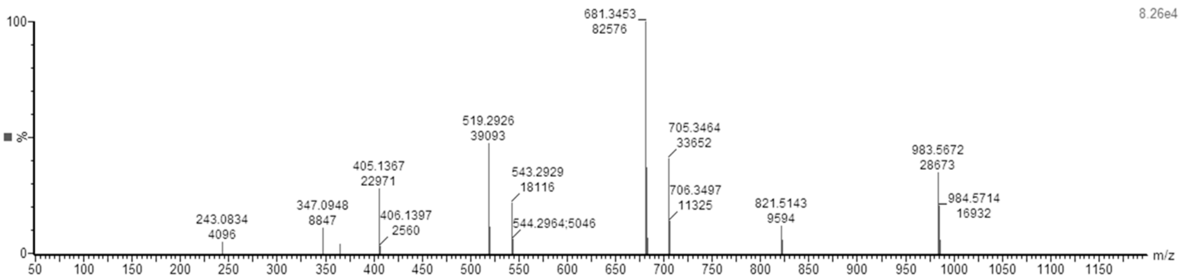

Figure S20. Chemical structures of annotated metabolites in the *F. vesiculosus n*-BuOH extract. Numbers correspond to putatively annotated known compounds reported in Table S2

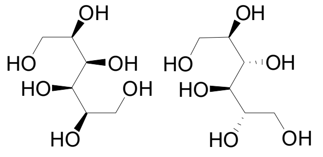

b1

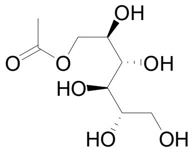

b2

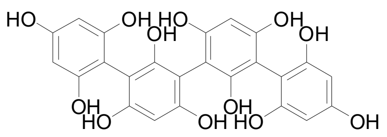

b3

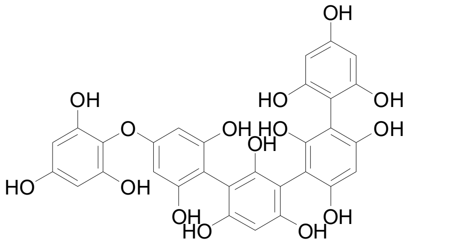

b4

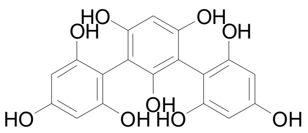

b5

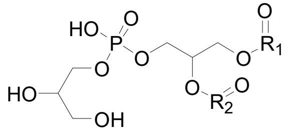

b10 - R1 + R2 C(32:1)
b12 - R1 + R2 C(32:0)

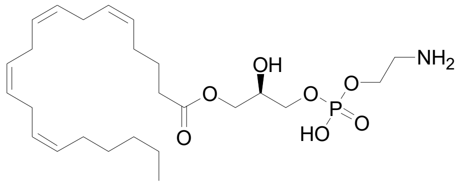

b16

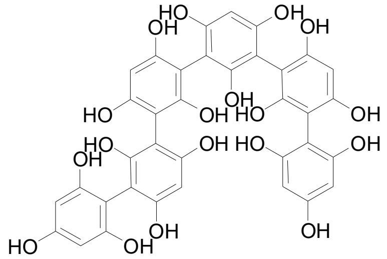

b6

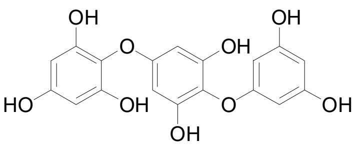

b7

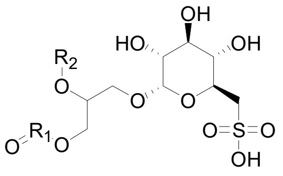

b11 - R_1 C(18:3); R_2 OC(14:0)
b13 - R_1 C(18:2); R_2 OC(14:0)
b14 - R_1 C(14:0); R_2 H
b15 - R_1 C(18:3); R_2 H
b17 - R_1 C(16.0); R_2 H

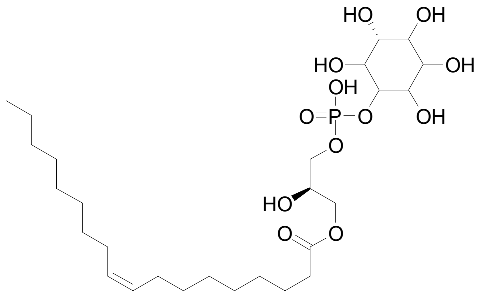

b18

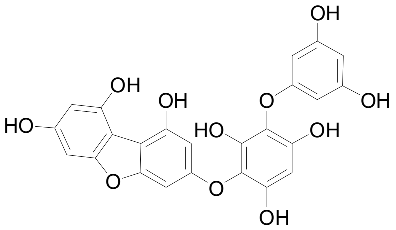

b8

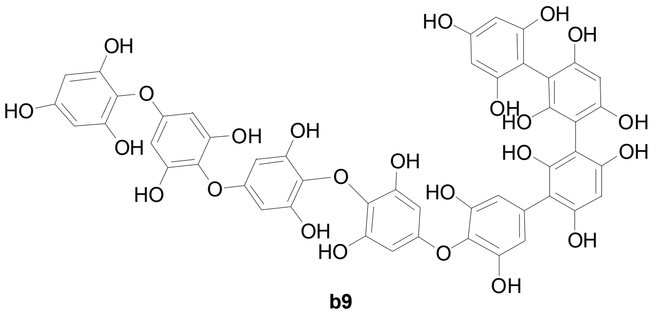

b9

Figure 21. GNPS MS/MS mirror plot of experimental and library data of mannitol (annotation **b1**)

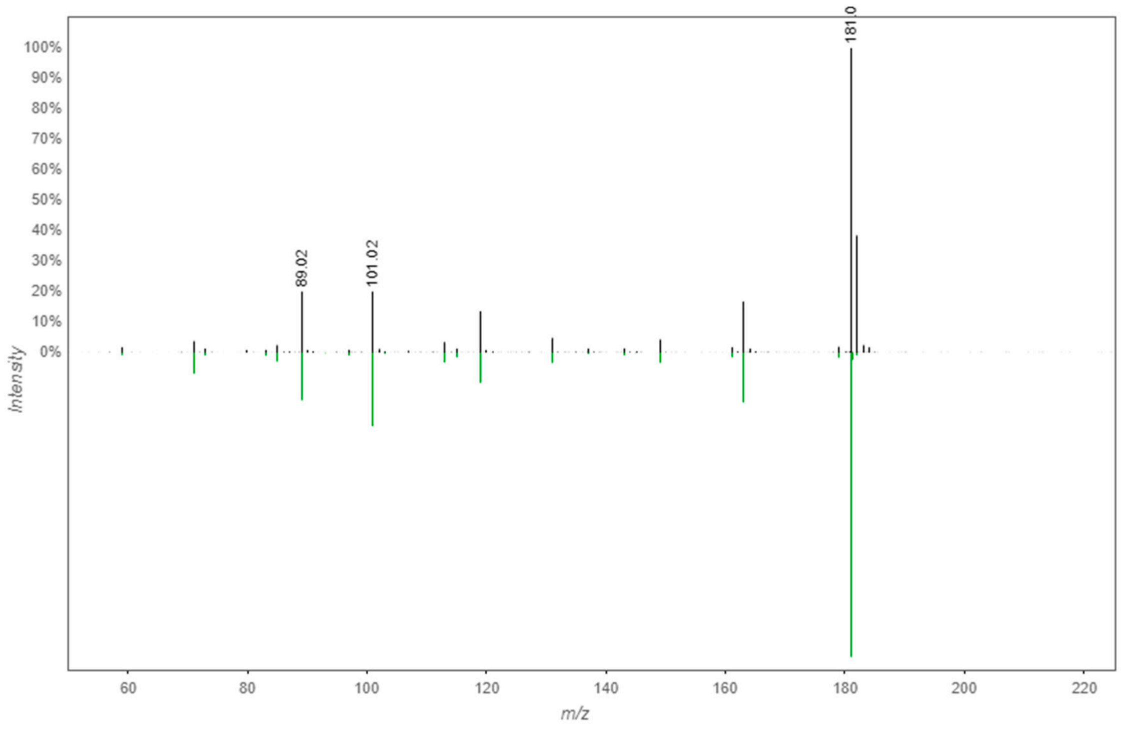

Figure 22. GNPS MS/MS mirror plot of experimental and library data of 1-(5Z,8Z,11Z,14Z-eicosatetraenoyl)-*sn*-glycero-3-phospho-ethanolamine (annotation **b16)**

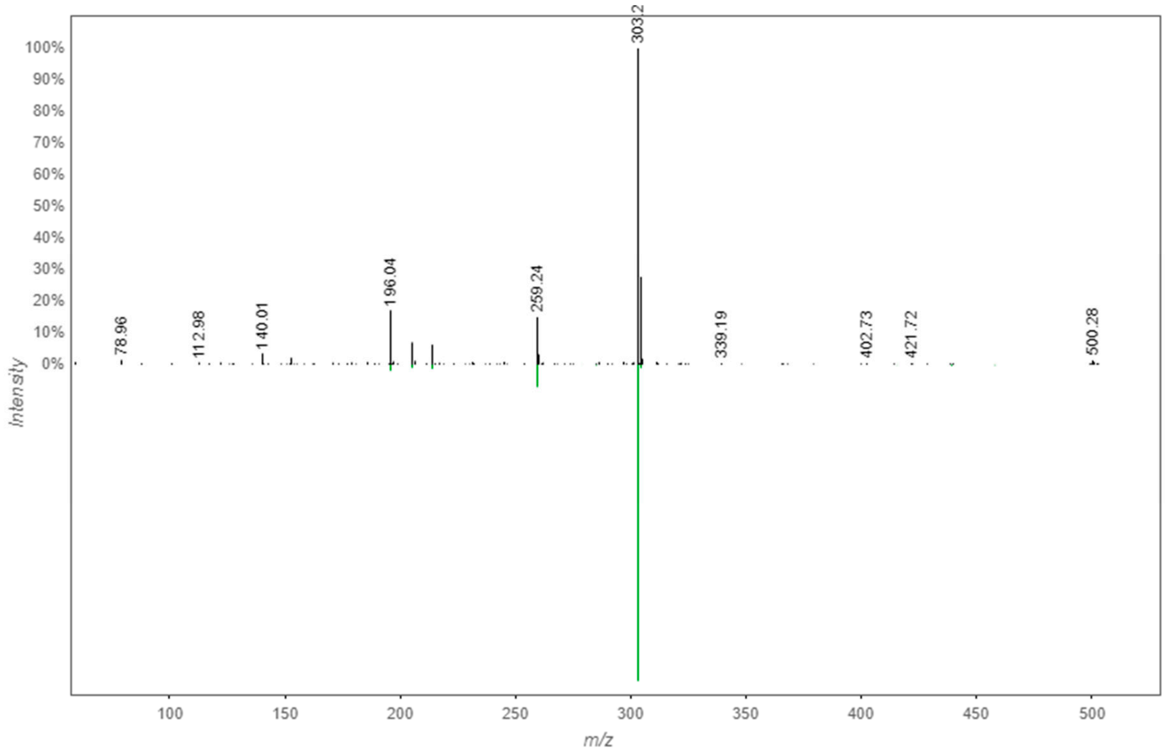

Figure S23. HR-ESI-MS and MS/MS spectra of compound **8**.

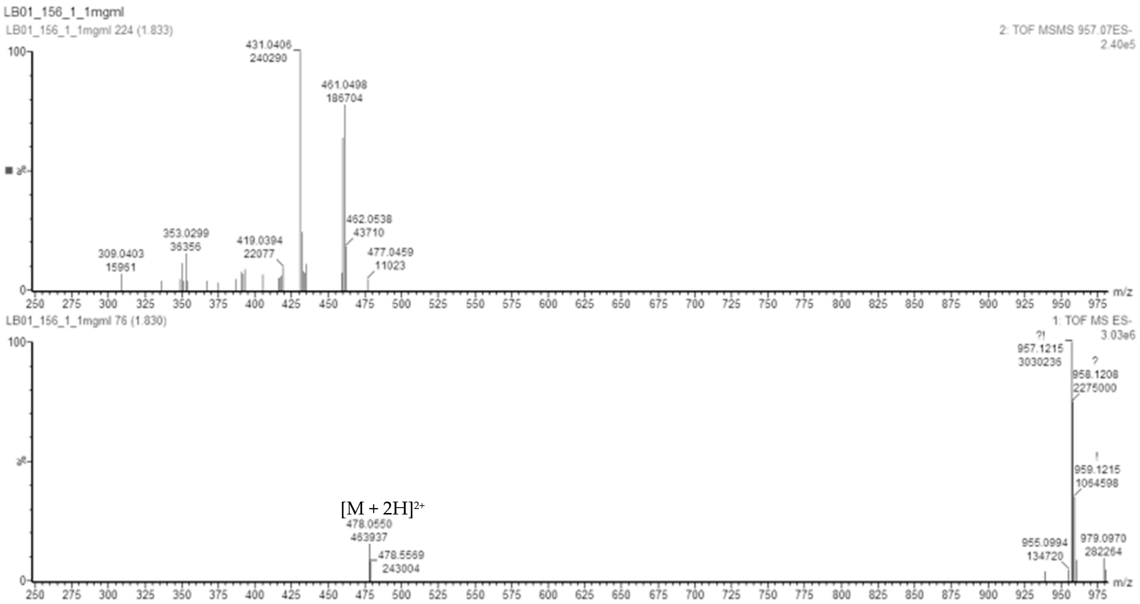

Figure S24. ^{1}H NMR spectrum of compound **8** (DMSO-d_6, 600 MHz).

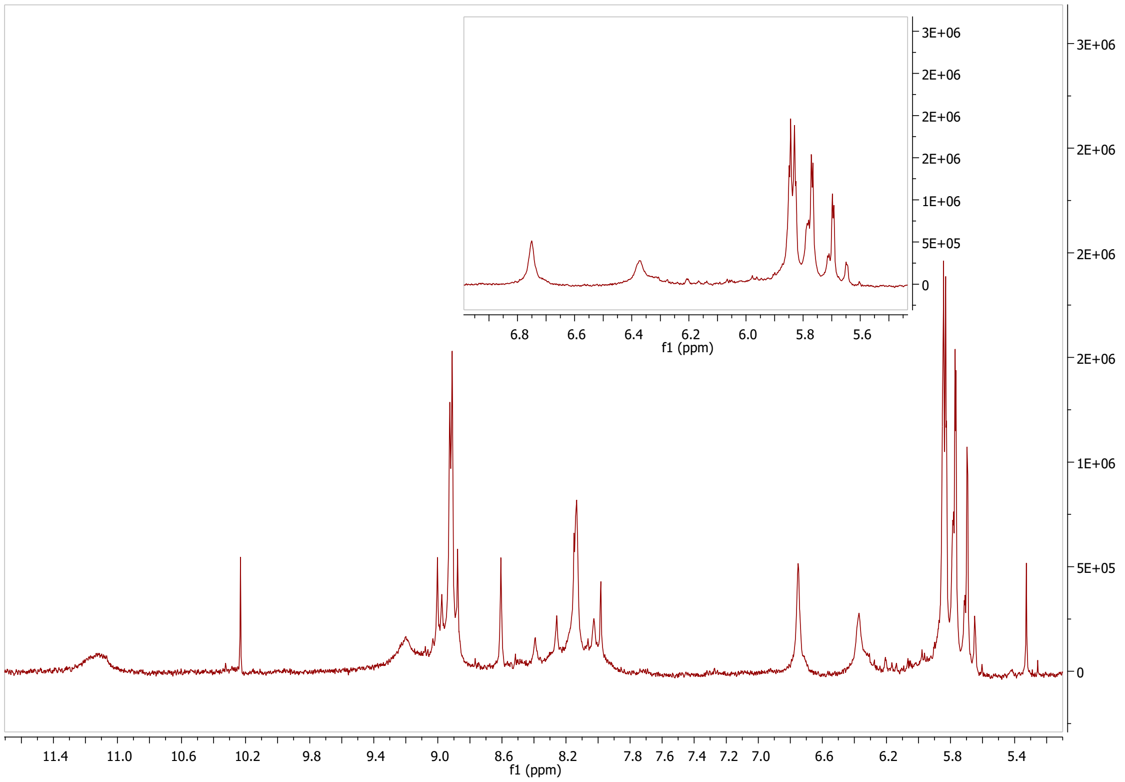

Figure S25. COSY spectrum of compound **8** (DMSO-*d*$_6$, 600 MHz).

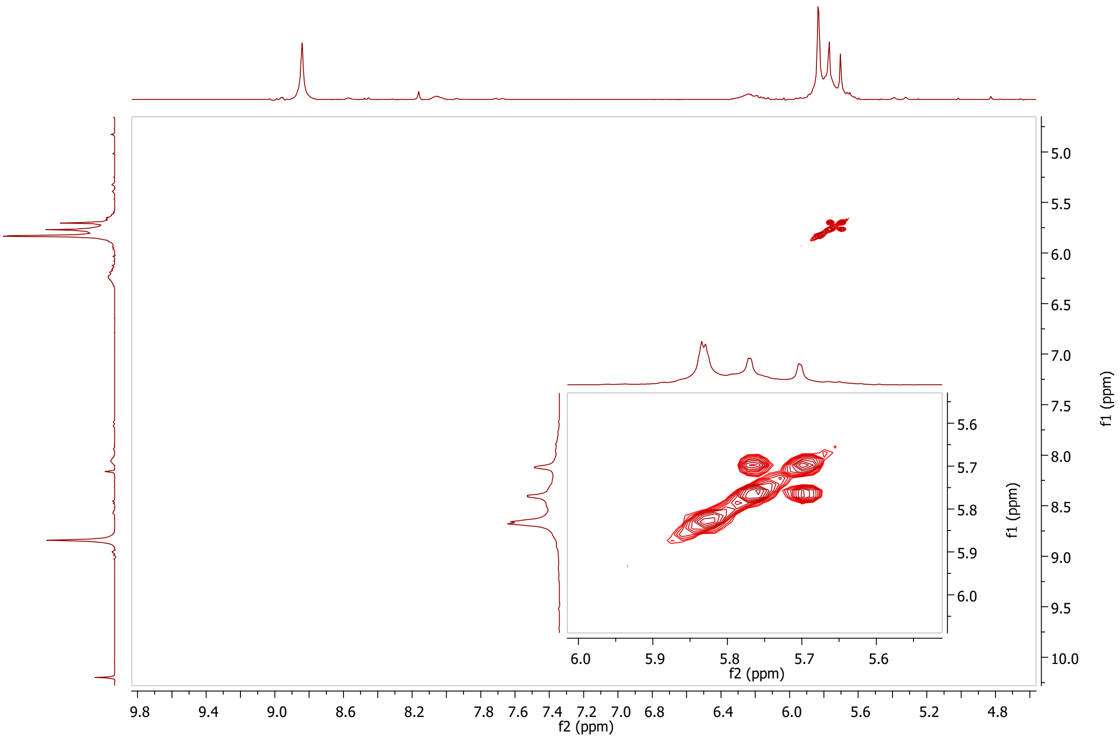

Figure S26. HSQC spectrum of compound **8** (DMSO-*d*$_6$, 600 MHz).

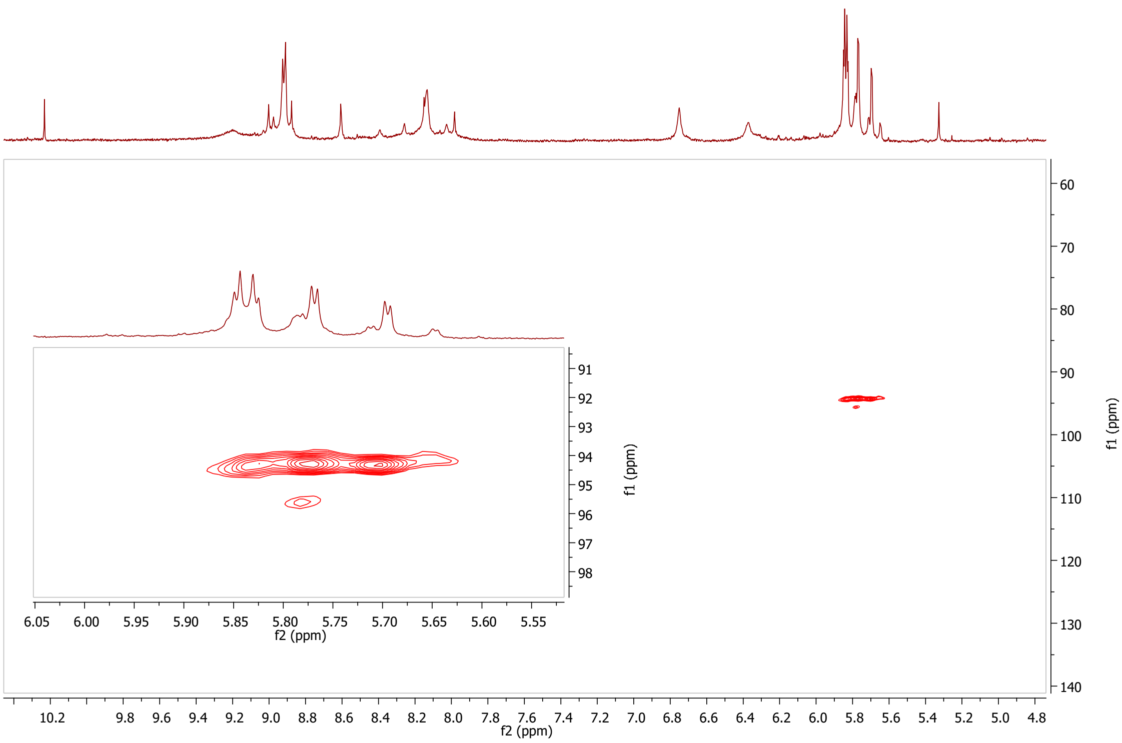

Figure S27. HMBC spectrum of compound **8** (DMSO-*d*$_6$, 600 MHz).

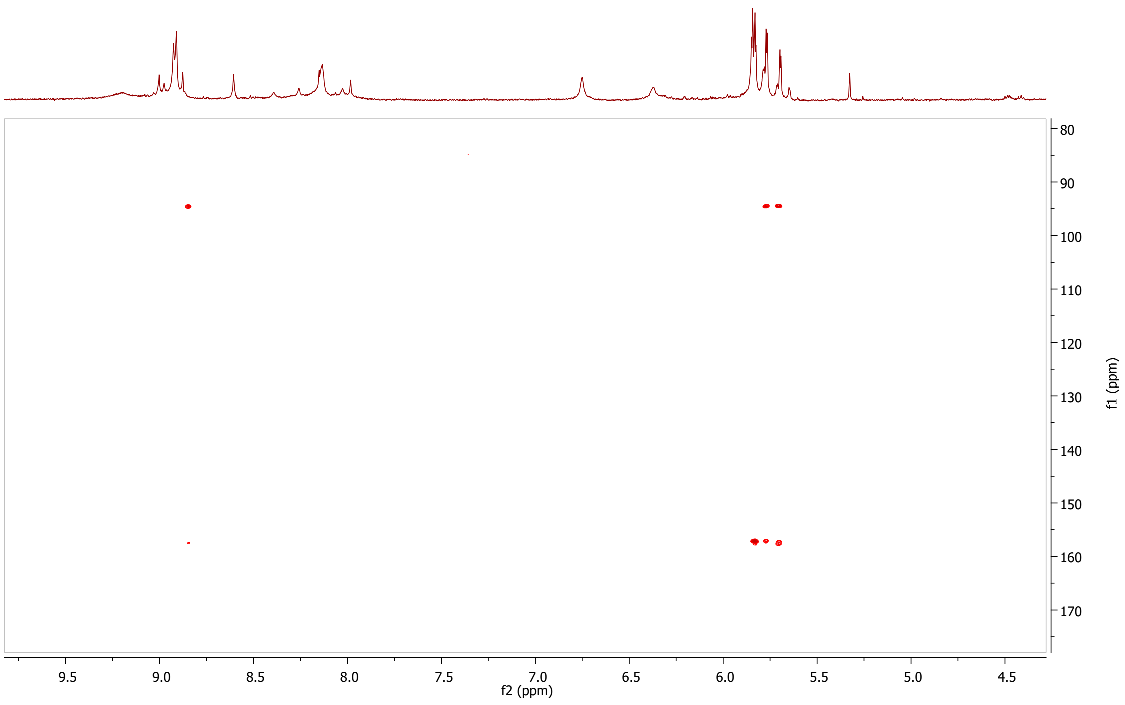

Figure S28. HR-ESI-MS spectrum of **8** showing characteristic isotopic pattern of hydrogen-deuterium exchange.

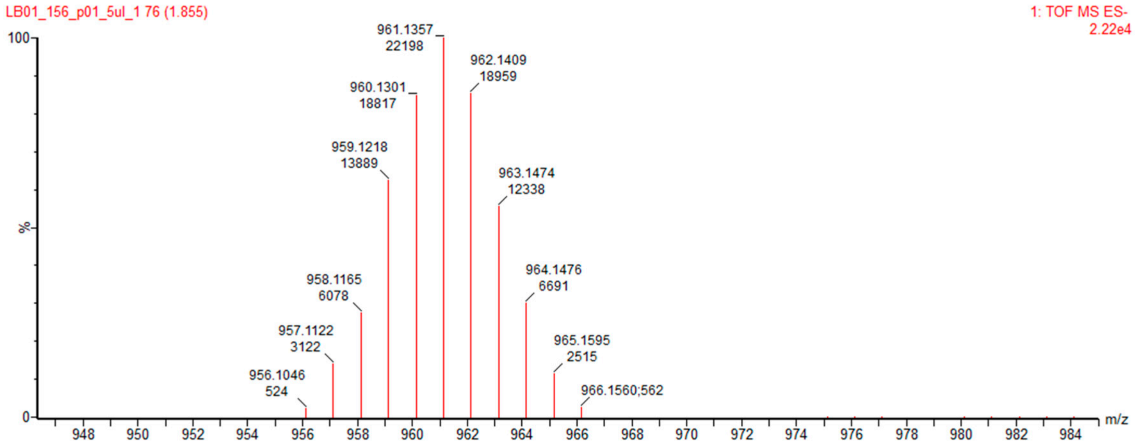

Figure S29. HR-ESI-MS/MS spectrum of **9**.

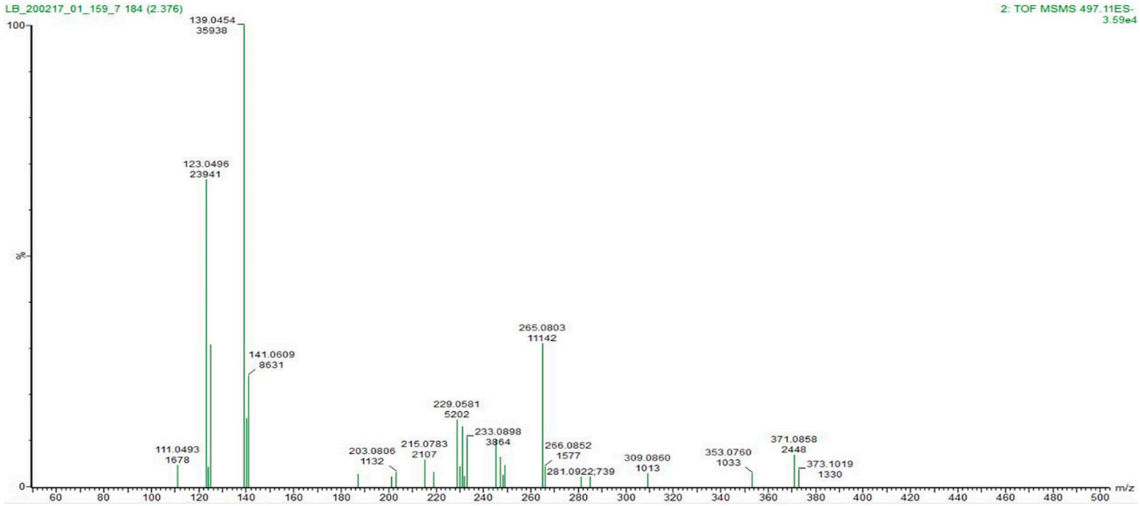

References

1. Sumner, L.W.; Amberg, A.; Barrett, D.; Beale, M.H.; Beger, R.; Daykin, C.A.; Fan, T.W.-M.; Fiehn, O.; Goodacre, R.; Griffin, J.L. *et al.* Proposed minimum reporting standards for chemical analysis. *Metabolomics* **2007**, *3*, 211-221. https://doi.org/10.1007/s11306-007-0082-2
2. Blaženović, I.; Kind, T.; Ji, J.; Fiehn, O. Software tools and approaches for compound identification of LC-MS/MS data in metabolomics. *Metabolites* **2018**, *8*, 31. https://doi.org/10.3390/metabo8020031
3. Imbs, T.I.; Ermakova, S.P.; Fedoreyev, S.A.; Anastyuk, S.D.; Zvyagintseva, T.N. Isolation of fucoxanthin and highly unsaturated monogalactosyldiacylglycerol from brown alga *Fucus evanescens* C Agardh and *in vitro* investigation of their antitumor activity. *Mar. Biotechnol.* **2013**, *15*, 606-612. https://doi.org/10.1007/s10126-013-9507-2
4. Hong, C.-O.; Nam, M.-H.; Oh, J.-S.; Lee, J.-W.; Kim, C.-T.; Park, K.-W.; Lee, D.-H.; Lee, K.-W. Pheophorbide a from *Capsosiphon fulvescens* inhibits advanced glycation end products mediated endothelial dysfunction. *Planta Med.* **2016**, *82*, 46-57.
5. Da Costa, E.; Domingues, P.; Melo, T.; Coelho, E.; Pereira, R.; Calado, R.; Abreu, M.H.; Domingues, M.R. Lipidomic signatures reveal seasonal shifts on the relative abundance of high-valued lipids from the brown algae *Fucus vesiculosus*. *Mar. Drugs* **2019**, *17*, 335. https://doi.org/10.3390/md17060335
6. Newton, R.; Pennock, J. The intracellular distribution of tocopherols in plants. *Phytochemistry* **1971**, *10*, 2323-2328. https://doi.org/10.1016/S0031-9422(00)89873-8
7. Bilan, M.I.; Grachev, A.A.; Shashkov, A.S.; Nifantiev, N.E.; Usov, A.I. Structure of a fucoidan from the brown seaweed *Fucus serratus* L. *Carbohydr. Res.* **2006**, *341*, 238-245. https://doi.org/10.1016/j.carres.2005.11.009
8. Catarino, M.D.; Silva, A.; Mateus, N.; Cardoso, S.M. Optimization of phlorotannins extraction from *Fucus vesiculosus* and evaluation of their potential to prevent metabolic disorders. *Mar. Drugs* **2019**, *17*, 162. https://doi.org/10.3390/md17030162
9. Glombitza, K.-W.; Zieprath, G. Phlorotannins from the brown alga *Analipus japonicus*. *Planta Med.* **1989**, *55*, 171-175. DOI: 10.1055/s-2006-961916
10. Lopes, G.; Barbosa, M.; Vallejo, F.; Gil-Izquierdo, Á.; Andrade, P.B.; Valentão, P.; Pereira, D.M.; Ferreres, F. Profiling phlorotannins from *Fucus* spp. of the Northern Portuguese coastline: Chemical approach by HPLC-DAD-ESI/MS^n and UPLC-ESI-QTOF/MS. *Algal Res.* **2018**, *29*, 113-120. https://doi.org/10.1016/j.algal.2017.11.025
11. Kang, K.A.; Lee, K.H.; Chae, S.; Koh, Y.S.; Yoo, B.-S.; Kim, J.H.; Ham, Y.M.; Baik, J.S.; Lee, N.H.; Hyun, J.W. Triphlorethol-A from *Ecklonia cava* protects V79-4 lung fibroblast against hydrogen peroxide induced cell damage. *Free Radic. Res.* **2005**, *39*, 883-892. https://doi.org/10.1080/10715760500161165
